# Checklist of aquatic Diptera (Insecta) of Plitvice Lakes National Park, Croatia, a UNESCO world heritage site

**DOI:** 10.3897/zookeys.918.49648

**Published:** 2020-03-12

**Authors:** Marija Ivković, Valentina Dorić, Viktor Baranov, Zlatko Mihaljević, Levente-Péter Kolcsár, Gunnar Mikalsen Kvifte, Jana Nerudova, Adrian C. Pont

**Affiliations:** 1 Division of Zoology, Department of Biology, Faculty of Science, University of Zagreb, Rooseveltov trg 6, 10000 Zagreb, Croatia University of Zagreb Zagreb Croatia; 2 Eko-monitoring d.o.o., Kučanska 15, 42000 Varaždin, Croatia Eko-monitoring d.o.o. Varaždin Croatia; 3 LMU Munich Biocenter, Department of Biology II, Großhaderner Str. 2, 82152 Planegg-Martinsried, Germany LMU Munich Biocenter Planegg-Martinsried Germany; 4 Department of Civil and Environmental Engineering, Ehime University Bunkyo-cho 3, Matsuyama, 790-8577, Japan Ehime University Matsuyama Japan; 5 Department of Arts and Education, Nord University, P.O. Box 1490, 8049 Bodø, Norway Nord University Bodø Norway; 6 Moravian Museum, Department of Entomology, Hviezdoslavova 29a, 627 00 Brno, Czech Republic Moravian Museum, Department of Entomology Brno Czech Republic; 7 Oxford University Museum of Natural History, Parks Road, Oxford OX1 3PW, United Kingdom Oxford University Museum of Natural History Oxford United Kingdom

**Keywords:** Barrage lake system, Chironomidae, Empididae, Limoniidae, new records, *Oxycera* spp.

## Abstract

Studies on aquatic Diptera in the Plitvice Lakes National Park (Croatia) conducted in the last 50 years have produced 157 species and 7 taxa of aquatic Diptera placed in 13 families. Samples were collected at 25 sampling sites representing the four main types of karst aquatic habitats: spring, stream, tufa barriers and lakes. All records of all the aquatic families of Diptera in Plitvice Lakes NP are summarized, including previously unpublished data. Twelve species new for Plitvice Lakes NP are recorded for the first time, belonging to the families: Chironomidae – *Labrundinia longipalpis* (Goetghebuer, 1921), *Nilothauma brayi* (Goetghebuer, 1921), *Potthastia longimanus* Kieffer, 1922, Polypedilum (Polypedilum) nubeculosum (Meigen, 1804), *Tanytarsus brundini* Lindeberg, 1963; Dixidae – *Dixella autumnalis* (Meigen, 1838); Scathophagidae – *Acanthocnema latipennis* Becker, 1894 and Stratiomyidae – *Oxycera pardalina* Meigen, 1822, *Oxycera limbata* Loew, 1862, *Oxycera turcica* Ustuner & Hasbenli, 2004, *Nemotelus pantherinus* (Linnaeus, 1758), *Oplodontha viridula* (Fabricius, 1775). The most species-rich family was the Chironomidae with 62 species (and an additional seven taxa), followed by the Empididae with 22 species and Limoniidae with 19 species. The highest number of species was recorded in springs. The relatively low number of species in certain families and the complete absence of some aquatic families shows that further research into the aquatic Diptera in Plitvice Lakes NP is needed.

## Introduction

Most people probably know true flies (Diptera) mainly as a nuisance and as disease-carrying blood-sucking insects, but Diptera are also key players in the recycling of organic material in ecosystems, from the sewage of our urban communities to the leaf litter of the forest floor. In addition, Diptera provide other general ecosystem services such as pollination and pest control, but are also vectors of disease as terrestrial adults (Pape 2009; Marshall 2012; Adler and Courtney 2019).

More than any other group of macro-organisms, Diptera dominate the freshwater environment and are the most numerous group in terms of described species in freshwaters. Nearly one-third of all described fly species, roughly 46,000 species, have some connection with an aquatic environment during development process (Adler and Courtney 2019). Their abundance, omnipresence, and diversity of adaptations to the aquatic environment, position them as major drivers of ecosystem processes (Hölker et al. 2015). Fly larvae are well represented as ecosystem engineers and keystone species that alter the abiotic and biotic environments through activities such as burrowing, grazing, suspension feeding, and predation (Wotton et al. 1998; Adler and Courtney 2019). The enormous populations sometimes achieved by aquatic flies can provide the sole or major dietary component for other organisms, and as both predators and herbivores, they can serve as biological control agents (Collins and Blackwell 2000; Werner and Pont 2003; Adler and Courtney 2019). They serve as indicators of historical and future ecological and climate change while at the same time they have played a role as indicators of water quality from the earliest years of bioassessment (Walker 1987; Mihaljević et al. 1998; Larocque et al. 2001; Adler and Courtney 2019).

As holometabolous insects that undergo complete metamorphosis, all aquatic Diptera have a life cycle that includes a series of distinct stages or instars. A typical life cycle consists of a brief egg stage (usually a few days or weeks, but sometimes much longer), three or four larval instars (usually three in Brachycera, four in lower Diptera, and more in Simuliidae, Tabanidae, Thaumaleidae, some Chironomidae, and a few others), a pupal stage of varying length, and an adult stage lasting from less than two hours (Deuterophlebiidae) to several weeks or even months (Courtney et al. 2017; Lackmann and Butler 2018; Adler and Courtney 2019).

From all types of aquatic habitats, including tree holes to open oceans, and glacial meltwaters to hot springs, Diptera are the true conquerors of the aquatic environment. They have been found at elevations up to 5600 m in the Himalayas and at depths of more than 1000 m in Lake Baikal. Furthermore, the presence of Diptera species in mainland Antarctica (e.g., *Belgica antarctica* Jacobs, 1900) makes them the only group of insects inhabiting all of the world’s continents (Allegrucci et al. 2006; Ferrington et al. 2008; Adler and Courtney 2019). Aquatic Diptera are free-living insects that require a wet environment in at least one life stage (Adler and Courtney 2019) or, more strictly, aquatic Diptera are considered as those associated with water bodies (Courtney et al. 2017). Out of 158 dipteran families worldwide, 41 have aquatic representatives (Adler and Courtney 2019), and in Europe there are 130 dipteran families of which about 25 are related to aquatic habitats (Oosterbroek 2006).

Plitvice Lakes form the oldest National Park in the Balkan region and is probably one of the most famous National Parks in Europe because of its exquisite beauty. Plitvice Lakes NP was established as a National Park in 1949, and from 1979 Plitvice lakes NP has been a UNESCO world natural heritage site (Stilinović and Božičević 1998). Its importance is not only scientific, as a unique karstic phenomenon, but also as a place of huge economic importance for the local community as more than a million people per year come to visit it.

## Materials and methods

### Study site

Plitvice Lakes National Park (NP) is a 295 km^2^ forest reserve located in the karst region of the Dinaric Mountains in Croatia. The Plitvice Lakes barrage lake system consists of 16 oligotrophic, dimictic and fluvial lakes divided by tufa barriers that form an approximately 8.2 km long barrage system. The lakes are characterised by a low organic solute concentration, supersaturation with calcium salts, pH > 8.0, and the presence of algae and mosses mediating tufa barriers formation (Srdoč et al. 1985; Stilinović and Božičević 1998). After the confluence of the Bijela rijeka and Crna rijeka Rivers, they form the Matica River which is the main surface-water supplier of the lakes (Stilinović and Božičević 1998). According to the Köppen climate classification, this area is influenced by temperate and boreal climates (Šegota and Filipčić 2003).

### Specimen records

**Figure 1.**
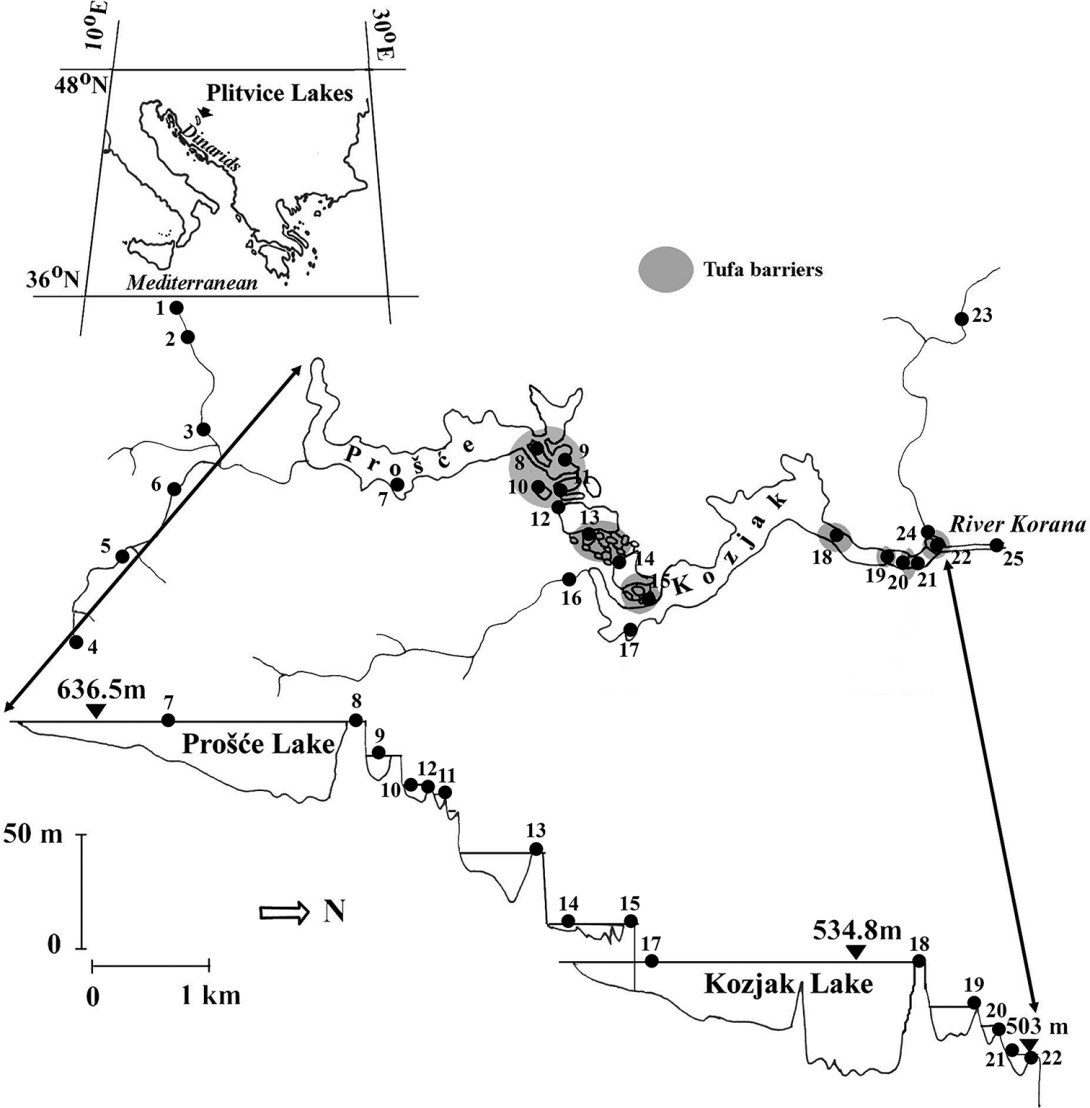
Sampling sites in Plitvice Lakes National Park (see Table 1 for codes).

**Figure 2.**
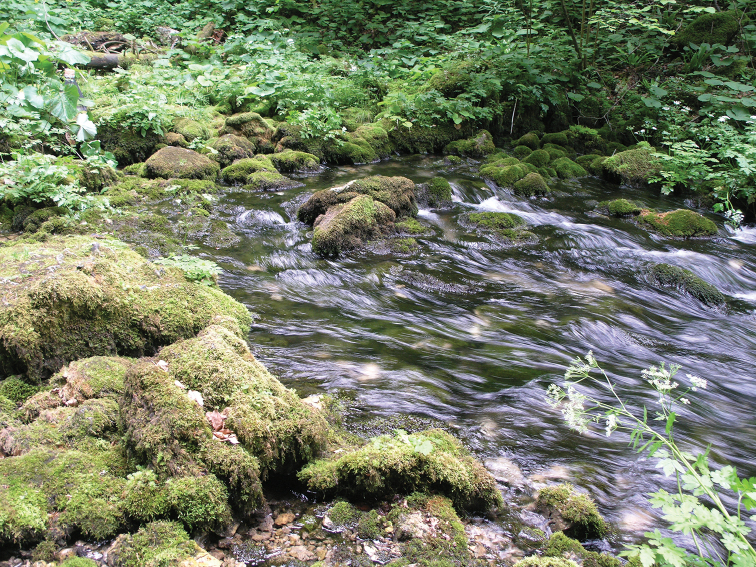
Spring of Crna Rijeka, Plitvice Lakes National Park.

**Figure 3.**
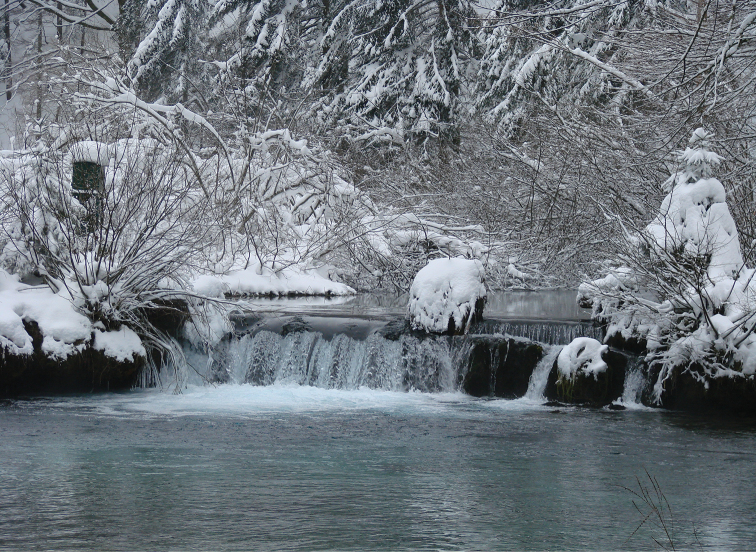
Crna Rijeka by the bridge, Plitvice Lakes National Park.

**Figure 4.**
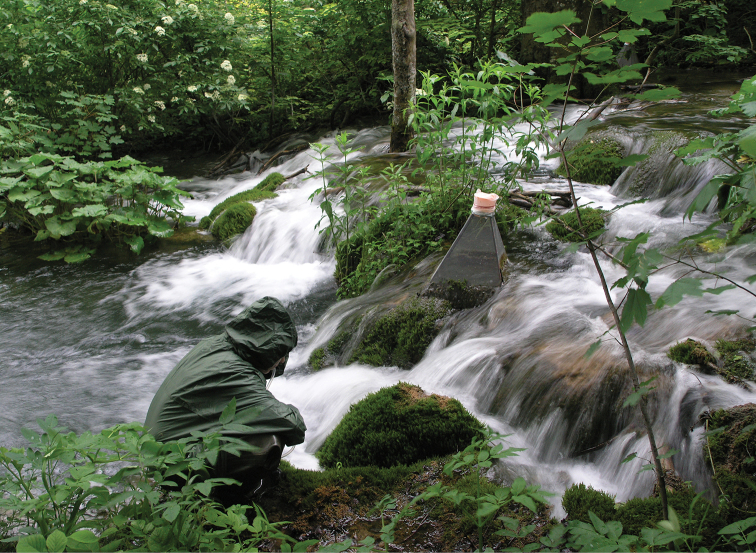
Tufa barrier Labudovac, Plitvice Lakes National Park.

**Figure 5.**
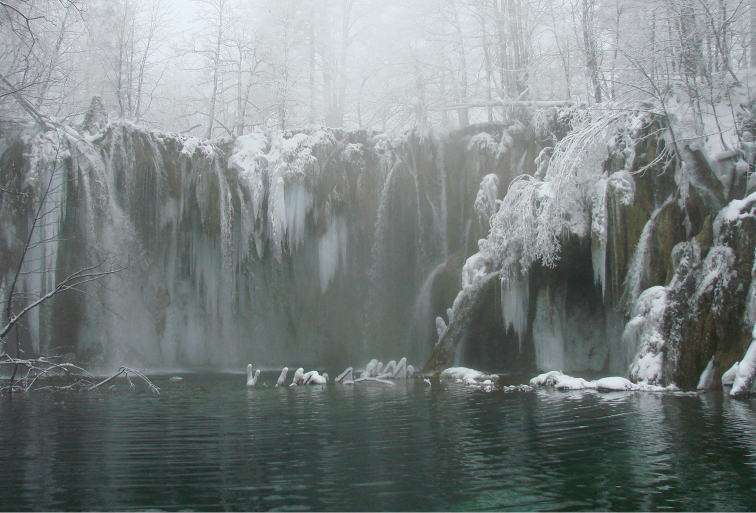
Tufa barrier Galovac-Milino, Plitvice Lakes National Park.

**Figure 6.**
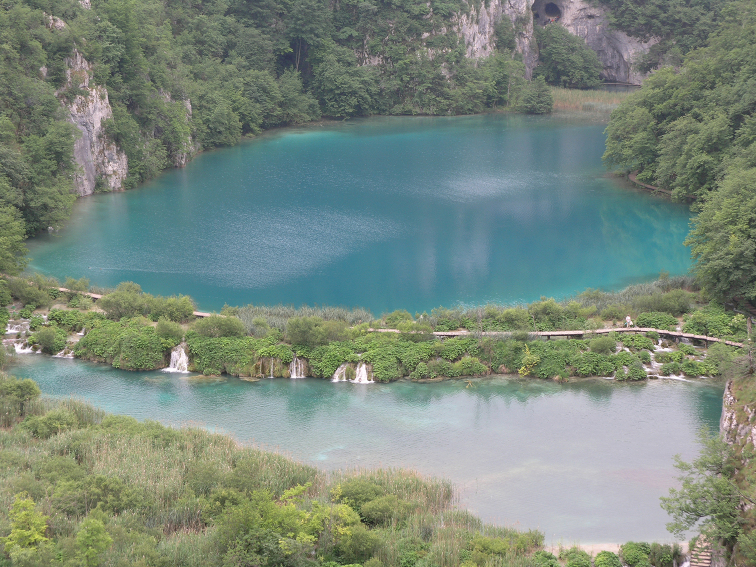
Lake Kaluđerovac and Tufa barrier Novakovića Brod, Plitvice Lakes National Park.

**Figure 7.**
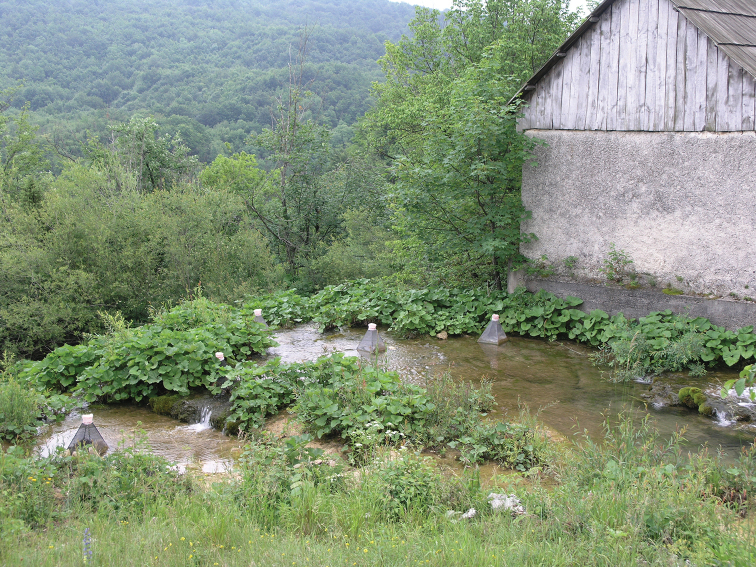
Stream Plitvica, Plitvice Lakes National Park.

This paper is based on unpublished data from our own research and on published data gleaned from the literature. Each record was georeferenced using ArcGIS software. The names of taxa presented in this checklist reflect current nomenclature and classifications (Yang et al. 2007; Ashe and O’Connor 2009, 2012; Pape and Beuk 2012; Adler and Crosskey 2018; Ivković et al. 2019; Oosterbroek 2019; Starý 2019). Locality records are listed for each species. A list of locality names including latitude, longitude, altitude, and number code for each locality is given in Table 1, and a map with all sites plotted is provided as Figure 1. Photographs of several studied sites are also given (Figs 2–7). Adult specimens were collected using emergence traps (details in Ivković et al. 2013a), sweep nets, yellow pan traps and aspirators, whereas larvae were collected by Surber sampler (25 × 25 cm) and kick-net sampler (25 × 25 cm, 500 µm mesh size). Specimens were preserved in 80% or 96% ethanol (EtOH). We identified the specimens to species level using Thomas (1997) for Athericidae; Reiss and Fittkau (1971); Bitušík (2000), Langton and Pinder (2007a, 2007b), Andersen et al. (2013), Bitušík and Hamerlík (2014), Vallenduuk (2017) for Chironomidae; Disney (1999) for Dixidae; Engel (1938–1946) for Empididae; Gorodkov (1988) for Scathophagidae; and Rozkošný and Kniepert (2000) for Stratiomyidae.

**Table 1.** Sampling sites in National Park Plitvice Lakes, Croatia.

Site Name	Site ID	Latitude / Longitude	Elevation (m)
Spring of Bijela rijeka, Plitvice Lakes NP	1	44°49'58"N, 15°33'25"E	720
Upper reach of Bijela rijeka, Plitvice Lakes NP	2	44°50'04"N, 15°33'33"E	715
Plitvički Ljeskovac, Plitvice Lakes NP	3	44°50'27"N, 15°35'40"E	668
Spring of Crna rijeka, Plitvice Lakes NP	4	44°50'14"N, 15°36'28"E	680
Upper reach of Crna rijeka, Plitvice Lakes NP	5	44°50'10"N, 15°36'30"E	670
Crna rijeka by the bridge, Plitvice Lakes NP	6	44°50'22"N, 15°35'59"E	665
Lake Prošće, Plitvice Lakes NP	7	44°51'33"N, 15°36'09"E	635
Tufa barrier Labudovac, Plitvice Lakes NP	8	44°52'17"N, 15°35'59"E	630
Lake Okrugljak, Plitvice Lakes NP	9	44°52'23"N, 15°35'56"E	626
Lake Batinovac, Plitvice Lakes NP	10	44°52'16"N, 15°36'11"E	624
Tufa barrier Batinovac-Crno Lake-Malo Lake-Vir, Plitvice Lakes NP	11	44°52'25"N, 15°36'10"E	603
Tufa barrier Batinovac-Galovac, Plitvice Lakes NP	12	44°52'21"N, 15°36'15"E	605
Tufa barrier Galovac-Milino, Plitvice Lakes NP	13	44°52'32"N, 15°36'29"E	594
Lake Gradinsko, Plitvice Lakes NP	14	44°52'39"N, 15°36'37"E	565
Tufa barrier Burget-Kozjak, Plitvice Lakes NP	15	44°52'47"N, 15°36'53"E	547
Riječica, Plitvice Lakes NP	16	44°52'27"N, 15°36'47"E	555
Lake Kozjak, Plitvice Lakes NP	17	44°52'40"N, 15°37'07"E	535
Tufa barrier Kozjak-Milanovac,Plitvice Lakes NP	18	44°53'39"N, 15°36'32"E	545
Tufa barrier Milke Trnine, Plitvice Lakes NP	19	44°53'53"N, 15°36'39"E	540
Tufa barrier Gavanovac-Kaluđerovac, Plitvice Lakes NP	20	44°53'58"N, 15°36'39"E	537
Lake Kaluđerovac, Plitvice Lakes NP	21	44°54'02"N, 15°36'40"E	535
Tufa barrier Novakovića Brod, Plitvice Lakes NP	22	44°54'08"N, 15°36'38"E	505
Stream Sartuk, Plitvice Lakes NP	23	44⁰55'57"N, 15⁰33'10"E	765
Stream Plitvica, Plitvice Lakes NP	24	44°54'08"N, 15°36'27"E	555
Korana Village, Plitvice Lakes NP	25	44°55'33"N, 15°37'09"E	390

## Results and discussion

### List of aquatic Diptera of National Park Plitvice Lakes

The following format is used for the distribution data: literature references (name of the site and in parentheses the citation of site ID and the reference); new records (life stage in which the identifications were made, i.e., adult ♂, ♀ and larvae, name of the site and in parentheses the site ID, date of collection and the collector if possible). New species for Plitvice Lakes NP are listed with an asterisk before the name of the species. All the sites and site ID are listed in Table 1.

#### Family Athericidae

##### Ibisia marginata

Taxon classificationAnimaliaDipteraAthericidae

(Fabricius, 1781)

87CDF707-0E08-5F6B-A99C-2D9E7130BA9F

###### Literature reference.

• tufa barrier Burget-Kozjak, Plitvice Lakes NP (15) (Sertić Perić et al. 2014).

###### New records.

• 1♂; tufa barrier Labudovac, Plitvice Lakes NP (8); 26 Jul. 2010; M. Ivković leg. • 1♂; tufa barrier Kozjak-Milanovac, Plitvice Lakes NP (18); 28 Jun. 2012; M. Ivković leg. • 1♀; same site; 25 Jul. 2014; M. Ivković leg. • 3♂, 6♀; tufa barrier Novakovića Brod, Plitvice Lakes NP (22); 25 Jul. 2007; M. Ivković leg. • 10♂, 21♀; same site; 30 Aug. 2007; M. Ivković leg. • 1♂, 6♀; Korana Village, Plitvice Lakes NP (25); 29 Aug. 2008; M. Ivković leg.

#### Family Chironomidae

##### Subfamily Chironominae

###### Cryptochironomus (Cryptochironomus) albofasciatus

Taxon classificationAnimaliaDipteraChironomidae

(Staeger, 1839)

C25483A9-BCAA-5756-A30C-26DCDA9C5869

####### Literature reference.

• Lake Prošće, Plitvice Lakes NP (7) • Lake Kozjak, Plitvice Lakes NP (17) (Kostić-Brnek and Brnek-Kostić 1971).

###### Dicrotendipes nervosus

Taxon classificationAnimaliaDipteraChironomidae

(Staeger, 1839)

BCA923D9-A870-5C06-AF15-E0DA80FF9BA9

####### Literature reference.

• Lake Prošće, Plitvice Lakes NP (7) • Lake Kozjak, Plitvice Lakes NP (17) (Kostić-Brnek and Brnek-Kostić 1971).

####### Remark.

Mentioned as *Limnochironomus nervosus* (Staeger, 1839) in Kostić-Brnek and Brnek-Kostić (1971), an accepted synonym of *D. nervosus* in Spies and Saether (2013).

###### Endochironomus gr. dispar sensu

Taxon classificationAnimaliaDipteraChironomidae

Moller Pillot, 2009

2C07335F-D686-55CD-BB7D-5ECB13DFCC1B

####### Literature reference.

• Lake Prošće, Plitvice Lakes NP (7) • Lake Kozjak, Plitvice Lakes NP (17) (Kostić-Brnek and Brnek-Kostić 1971).

###### Einfeldia dissidens

Taxon classificationAnimaliaDipteraChironomidae

(Walker, 1856)

AC71FAC3-99E1-50B8-A797-99456A8D11FE

####### Literature reference.

• Lake Prošće, Plitvice Lakes NP (7) • Lake Kozjak, Plitvice Lakes NP (17) (Kostić-Brnek and Brnek-Kostić 1971).

###### Harnischia fuscimanus

Taxon classificationAnimaliaDipteraChironomidae

Kieffer, 1921

73F2F850-B1DE-5D9E-BC00-694DE9D8BBAE

####### Literature reference.

• Lake Prošće, Plitvice Lakes NP (7) • Lake Kozjak, Plitvice Lakes NP (17) (Kostić-Brnek and Brnek-Kostić 1971).

####### Remark.

Mentioned as *Cryptochironomus fuscimanus* Kieffer, 1921 in Kostić-Brnek and Brnek-Kostić (1971) a synonym of *H. fuscimanus* in Moller Pillot (2009).

###### Micropsectra notescens

Taxon classificationAnimaliaDipteraChironomidae

(Walker, 1856)

0BF98A90-1F03-5FEC-A5AE-C237C98AEB17

####### Literature reference.

• spring of Bijela rijeka, Plitvice Lakes NP (1) • spring of Crna rijeka, Plitvice Lakes NP (4) (Ivković et al. 2015).

###### Micropsectra uva

Taxon classificationAnimaliaDipteraChironomidae

Giłka, Zakrzewska, Baranov & Dominiak, 2013

47831FF9-7B84-50AD-B9C9-FD7D21615E4E

####### Literature reference.

• spring of Bijela rijeka, Plitvice Lakes NP (1) (Giłka et al. 2013, Ivković et al. 2015) • spring of Crna rijeka, Plitvice Lakes NP (4) (Ivković et al. 2015).

###### Microtendipes pedellus

Taxon classificationAnimaliaDipteraChironomidae

(De Geer, 1776)

7AD95E4F-8FA6-5247-9EF2-86947B2F950D

####### Literature reference.

• Lake Prošće, Plitvice Lakes NP (7) • Lake Kozjak, Plitvice Lakes NP (17) (Kostić-Brnek and Brnek-Kostić 1971).

###### Microtendipes tarsalis

Taxon classificationAnimaliaDipteraChironomidae

(Walker, 1856)

9718CB8D-D206-5524-912B-796BE9086763

####### Literature reference.

• Lake Prošće, Plitvice Lakes NP (7) • Lake Kozjak, Plitvice Lakes NP (17) (Kostić-Brnek and Brnek-Kostić 1971).

###### * Nilothauma brayi

Taxon classificationAnimaliaDipteraChironomidae

(Goetghebuer, 1921)

4A527B62-F4C2-563B-AF64-2AF18BE0D2B2

####### New record.

• 1 larva; Lake Kozjak, Plitvice Lakes NP (17); 18 Jul. 2018.

###### Paracladopelma camptolabis

Taxon classificationAnimaliaDipteraChironomidae

(Kieffer, 1913)

26F507B8-80FD-5B5C-BE25-2DBDF991A4AE

####### Literature reference.

• Lake Prošće, Plitvice Lakes NP (7) • Lake Kozjak, Plitvice Lakes NP (17) (Kostić-Brnek and Brnek-Kostić 1971).

###### Paratanytarsus lauterborni

Taxon classificationAnimaliaDipteraChironomidae

(Kieffer, 1909)

9BE9F6E4-8671-5444-A1A7-1DBEFB5D0597

####### Literature reference.

• Lake Prošće, Plitvice Lakes NP (7) • Lake Kozjak, Plitvice Lakes NP (17) (Kostić-Brnek and Brnek-Kostić 1971).

###### Paratendipes albimanus

Taxon classificationAnimaliaDipteraChironomidae

(Meigen, 1818)

ED8B64D7-62CA-587E-9888-522E65705FBD

####### Literature reference.

• Lake Prošće, Plitvice Lakes NP (7) • Lake Kozjak, Plitvice Lakes NP (17) (Kostić-Brnek and Brnek-Kostić 1971).

###### Phaenopsectra flavipes

Taxon classificationAnimaliaDipteraChironomidae

(Meigen 1818)

60DD7E19-A4FD-5E1B-8675-71343BB0E986

####### Literature reference.

• spring of Crna rijeka, Plitvice Lakes NP (4) (Ivković et al. 2015).

####### New record.

• 24 larvae; Lake Kozjak, Plitvice Lakes NP (17); 18 Jul. 2018.

###### Polypedilum (Pentapedilum) exsectum

Taxon classificationAnimaliaDipteraChironomidae

(Kieffer, 1916)

874B0C86-3490-527C-9D24-7E69CCCF9C25

####### Literature reference.

• Lake Prošće, Plitvice Lakes NP (7) • Lake Kozjak, Plitvice Lakes NP (17) (Kostić-Brnek and Brnek-Kostić 1971).

####### Remark.

Mentioned as *Pentapedilum exsectum* Kieffer, 1913 in Kostić-Brnek and Brnek-Kostić (1971), an accepted synonym of *P. exsectum* in Spies and Saether (2013).

###### * Polypedilum (Polypedilum) nubeculosum

Taxon classificationAnimaliaDipteraChironomidae

(Meigen, 1804)

C5F9E341-88AA-502B-88E3-03CD1FCDF6B9

####### New record.

• 6 larvae; Lake Kozjak, Plitvice Lakes NP (17); 18 Jul. 2018.

###### Polypedilum (Tripodura) scalaenum

Taxon classificationAnimaliaDipteraChironomidae

(Schrank, 1803)

730F9836-9806-572D-BCE9-E750CA6A3CF5

####### Literature reference.

• Lake Prošće, Plitvice Lakes NP (7) • Lake Kozjak, Plitvice Lakes NP (17) (Kostić-Brnek and Brnek-Kostić 1971).

####### Remark.

Mentioned as *Polypedilum breviantennatum* Chernovskij, 1949 in Kostić-Brnek and Brnek-Kostić (1971), an accepted synonym of *P. scalaenum* in Spies and Saether (2013).

###### Rheotanytarsus nigricauda

Taxon classificationAnimaliaDipteraChironomidae

Fittkau, 1960

5DE20CEC-C723-5214-B07C-1A9BEA26EA8E

####### Literature reference.

• spring of Bijela rijeka, Plitvice Lakes NP (1) • spring of Crna rijeka, Plitvice Lakes NP (4) (Ivković et al. 2015).

###### Rheotanytarsus pentapoda

Taxon classificationAnimaliaDipteraChironomidae

(Kieffer, 1909)

D547F5D3-0DEA-53C1-82C9-D43E49C0E251

####### Literature references.

• tufa barrier Labudovac, Plitvice Lakes NP (8) • tufa barrier Galovac-Milino, Plitvice Lakes NP (13) • tufa barrier Burget-Kozjak, Plitvice Lakes NP (15) • tufa barrier Milke Trnine, Plitvice Lakes NP (19) (Matoničkin et al. 1971, Matoničkin 1987).

####### Remark.

Mentioned as *Rheotanytarsus lapidicola* Kieffer, 1909 in Matoničkin et al. (1971) and Matoničkin (1987), an accepted synonym of *R. pentapoda* in Spies and Saether (2013).

###### Stempellina bausei

Taxon classificationAnimaliaDipteraChironomidae

(Kieffer, 1911)

F11B51BB-293B-52C8-B92C-4DC42DA76E81

####### Literature references.

• Plitvički Ljeskovac, Plitvice Lakes NP (3) (Matoničkin 1987) • Lake Batinovac, Plitvice Lakes NP (10) (Matoničkin et al. 1971) • tufa barrier Batinovac-Crno Lake-Malo Lake-Vir, Plitvice Lakes NP (11) (Matoničkin 1987) • Lake Gradinsko, Plitvice Lakes NP (14) • tufa barrier Burget-Kozjak, Plitvice Lakes NP (15) (Matoničkin et al. 1971).

####### New records.

• 50 larvae; Lake Prošće, Plitvice Lakes NP (7); 17 Sep. 2018 • 92 larvae; Lake Kozjak, Plitvice Lakes NP (17); 18 Jul. 2018.

###### * Tanytarsus brundini

Taxon classificationAnimaliaDipteraChironomidae

Lindeberg, 1963

DA0AFD92-C2A7-5BEE-9D7F-416BE7EBB886

####### New record.

• 2♂; tufa barrier Kozjak-Milanovac, Plitvice Lakes NP (18); 30 Jun. 2009; M. Ivković leg.

###### Tanytarsus heusdensis

Taxon classificationAnimaliaDipteraChironomidae

Goetghebuer, 1923

D436D237-C01C-52DF-A202-90160E600DB8

####### Literature reference.

• Lake Prošće, Plitvice Lakes NP (7) • Lake Kozjak, Plitvice Lakes NP (17) (Kostić-Brnek and Brnek-Kostić 1971).

###### Zavrelia pentatoma

Taxon classificationAnimaliaDipteraChironomidae

Kieffer & Bause, 1913

4BD0BA3D-F63B-50A6-9ED0-883C2C302DF7

####### Literature reference.

• Lake Prošće, Plitvice Lakes NP (7) • Lake Kozjak, Plitvice Lakes NP (17) (Kostić-Brnek and Brnek-Kostić 1971).

###### Zavreliella marmorata

Taxon classificationAnimaliaDipteraChironomidae

(van der Wulp, 1859)

02FA1371-DEF8-503C-9AE3-FCE0AF32A705

####### Literature reference.

• Lake Prošće, Plitvice Lakes NP (7) • Lake Kozjak, Plitvice Lakes NP (17) (Kostić-Brnek and Brnek-Kostić 1971).

##### Subfamily Diamesinae

###### Diamesa (Diamesa) thomasi

Taxon classificationAnimaliaDipteraChironomidae

Serra-Tosio, 1970

EEA5D26F-D309-50CD-AB33-AC6FEA476C20

####### Literature reference.

• spring of Bijela rijeka, Plitvice Lakes NP (1) (Baranov et al. 2012, Ivković et al. 2015).

###### Diamesa (Diamesa) tonsa

Taxon classificationAnimaliaDipteraChironomidae

(Haliday in Walker, 1856)

0D33D5F2-6609-55AB-8E3F-4E603BECBD99

####### Literature reference.

• spring of Bijela rijeka, Plitvice Lakes NP (1) (Ivković et al. 2015).

###### * Potthastia longimanus

Taxon classificationAnimaliaDipteraChironomidae

Kieffer, 1922

70DA206E-DE7C-5969-9ABC-FDD235AE6287

####### New record.

• 1 larva; Lake Kozjak, Plitvice Lakes NP (17); 18 Jul. 2018.

##### Subfamily Prodiamesinae

###### Monodiamesa bathyphila

Taxon classificationAnimaliaDipteraChironomidae

(Kieffer, 1918)

90541D98-D274-526B-8B3C-CEE1D7E5867D

####### Literature reference.

• Lake Prošće, Plitvice Lakes NP (7) • Lake Kozjak, Plitvice Lakes NP (17) (Kostić-Brnek and Brnek-Kostić 1971).

###### Prodiamesa olivacea

Taxon classificationAnimaliaDipteraChironomidae

(Meigen, 1818)

D4FD825E-7CCF-527C-9639-0945F0A16FD9

####### Literature reference.

• spring of Bijela rijeka, Plitvice Lakes NP (1) (Ivković et al. 2015) • Lake Prošće, Plitvice Lakes NP (7) (Kostić-Brnek and Brnek-Kostić 1971) • tufa barrier Labudovac, Plitvice Lakes NP (8) (Matoničkin et al. 1971, Matoničkin 1987) • Lake Kozjak, Plitvice Lakes NP (17) (Kostić-Brnek and Brnek-Kostić 1971) • tufa barrier Gavanovac-Kaluđervoac, Plitvice Lakes NP (20) (Matoničkin 1987) • Lake Kaluđerovac, Plitvice Lakes NP (21) (Matoničkin et al. 1971).

##### Subfamily Orthocladiinae

###### Acricotopus lucens

Taxon classificationAnimaliaDipteraChironomidae

(Zetterstedt, 1850)

458FCB5C-CEF8-541C-BAE9-ACE4A26930B9

####### Literature reference.

• Lake Prošće, Plitvice Lakes NP (7) • Lake Kozjak, Plitvice Lakes NP (17) (Kostić-Brnek and Brnek-Kostić 1971).

####### Remark.

Mentioned as *Acricotopus lucidus* Brundin, 1949 in Kostić-Brnek and Brnek-Kostić (1971), mentioned as a synonym of *A. lucens* in Moller Pillot (2013).

###### Brillia bifida

Taxon classificationAnimaliaDipteraChironomidae

(Kieffer, 1909)

231CEDE8-8356-5C18-A778-E2381911B244

####### Literature reference.

• spring of Bijela rijeka, Plitvice Lakes NP (1) • spring of Crna rijeka, Plitvice Lakes NP (4) (Ivković et al. 2015).

###### Brillia longifurca

Taxon classificationAnimaliaDipteraChironomidae

Kieffer, 1921

B46F7BAB-4B89-56EE-9757-DA20D93E42B4

####### Literature reference.

• Lake Prošće, Plitvice Lakes NP (7) • Lake Kozjak, Plitvice Lakes NP (17) (Kostić-Brnek and Brnek-Kostić 1971).

###### Chaetocladius dentiforceps

Taxon classificationAnimaliaDipteraChironomidae

(Edwards, 1929)

63081F75-2A40-59E1-83AE-A14E1F2D0180

####### Literature reference.

• spring of Crna rijeka, Plitvice Lakes NP (4) (Ivković et al. 2015).

###### Chaetocladius melaleucus

Taxon classificationAnimaliaDipteraChironomidae

(Meigen, 1818)

10FE5732-0FD7-59B2-9E9A-943AD597B6B1

####### Literature reference.

• spring of Bijela rijeka, Plitvice Lakes NP (1) (Ivković et al. 2015).

###### Cricotopus (Cricotopus) bicinctus

Taxon classificationAnimaliaDipteraChironomidae

(Meigen, 1818)

E776E3FA-E8BE-5D24-9EFF-ECDC7D631BFD

####### Literature reference.

• Stream Plitvica, Plitvice Lakes NP (24) (Matoničkin, 1971).

####### Remark.

Mentioned as *Trichocladius bicinctus* (Meigen, 1818) in Kostić-Brnek and Brnek-Kostić (1971).

###### Cricotopus (Cricotopus) fuscus

Taxon classificationAnimaliaDipteraChironomidae

(Kieffer, 1909)

1DD3F320-4329-5ADB-A3CA-B7F02DD2DE55

####### Literature reference.

• Stream Plitvica, Plitvice Lakes NP (24) (Matoničkin, 1971).

####### Remark.

Mentioned as Cricotopus (Cricotopus) biformis Edwards, 1929 in Matoničkin et al. (1971), known as a questionable synonym of C. (Cricotopus) fuscus in Ashe and O’Connor (2012).

###### Corynoneura lobata

Taxon classificationAnimaliaDipteraChironomidae

Edwards, 1924

7EB70878-7EA5-5FD4-B886-7AFFB8D3B95D

####### Literature reference.

• spring of Bijela rijeka, Plitvice Lakes NP (1) • spring of Crna rijeka, Plitvice Lakes NP (4) (Ivković et al. 2015).

###### Eukiefferiella devonica

Taxon classificationAnimaliaDipteraChironomidae

(Edwards, 1929)

CDE12BFA-6694-5BFC-98E0-F1C746703CB9

####### Literature reference.

• spring of Bijela rijeka, Plitvice Lakes NP (1) • spring of Crna rijeka, Plitvice Lakes NP (4) (Ivković et al. 2015).

###### Eukiefferiella gracei

Taxon classificationAnimaliaDipteraChironomidae

(Edwards, 1929)

52582C76-0842-5086-8DF1-DC044BA68BE5

####### Literature reference.

• tufa barrier Labudovac, Plitvice Lakes NP (8) (Matoničkin et al. 1971).

####### Remark.

Mentioned as *Eukiefferiella longicalcar* Kieffer in Matoničkin et al. (1971), an accepted synonym of *E. gracei* in Spies and Saether (2013).

###### Eukiefferiella ilkleyensis

Taxon classificationAnimaliaDipteraChironomidae

(Edwards, 1929)

473BD6F5-44E8-5340-BECC-3393F8302A2F

####### Literature reference.

• Stream Plitvica, Plitvice Lakes NP (24) (Matoničkin 1987).

####### Remark.

Mentioned as *Plectrocladius eukiefferiella quadridentata* Chernovskij, 1949 in Matoničkin (1987); see Discussion for more details.

###### Eukiefferiella minor

Taxon classificationAnimaliaDipteraChironomidae

(Edwards, 1929)

AE2BDAB7-C0B2-55F8-A4E9-5F9E5F979C4C

####### Literature reference.

• spring of Bijela rijeka, Plitvice Lakes NP (1) (Ivković et al. 2015).

###### Epoicocladius ephemerae

Taxon classificationAnimaliaDipteraChironomidae

(Kieffer, 1924)

523D5A20-3738-5B3E-9D9C-83F78EB83B2D

####### Literature reference.

• Lake Prošće, Plitvice Lakes NP (7) • Lake Kozjak, Plitvice Lakes NP (17) (Kostić-Brnek and Brnek-Kostić 1971).

###### Heterotrissocladius marcidus

Taxon classificationAnimaliaDipteraChironomidae

(Walker, 1856)

4E67570B-C955-524E-AC43-A60F6AC9263D

####### Literature reference.

• Lake Prošće, Plitvice Lakes NP (7) • Lake Kozjak, Plitvice Lakes NP (17) (Kostić-Brnek and Brnek-Kostić 1971).

###### Limnophyes gurgicola

Taxon classificationAnimaliaDipteraChironomidae

(Edwards, 1929)

2EEFF656-B8CB-58B0-91CB-82E3BEDC3FBE

####### Literature reference.

• spring of Bijela rijeka, Plitvice Lakes NP (1) (Ivković et al. 2015).

###### Limnophyes cf. minimus sensu

Taxon classificationAnimaliaDipteraChironomidae

Langton & Pinder, 2007

86931162-9499-5280-94FB-6CD995301035

####### Literature reference.

• spring of Crna rijeka, Plitvice Lakes NP (4) (Ivković et al. 2015).

###### Metriocnemus cf. albolineatus sensu

Taxon classificationAnimaliaDipteraChironomidae

Langton & Pinder, 2007

A4809316-F30F-5C1E-A77B-1D06A1E5127A

####### Literature reference.

• spring of Crna rijeka, Plitvice Lakes NP (4) (Ivković et al. 2015).

###### Metriocnemus eurynothus

Taxon classificationAnimaliaDipteraChironomidae

(Holmgren, 1883)

ABC0FCDF-ADD6-5B24-86DC-E5EB09361848

####### Literature reference.

• spring of Bijela rijeka, Plitvice Lakes NP (1) (Ivković et al. 2015).

###### Metriocnemus intergerivus

Taxon classificationAnimaliaDipteraChironomidae

Sæther, 1995

8FD409FF-11CD-56D6-8E12-5C47A6610D37

####### Literature reference.

• spring of Bijela rijeka, Plitvice Lakes NP (1) (Ivković et al. 2015).

###### Orthocladius (Mesorthocladius) frigidus

Taxon classificationAnimaliaDipteraChironomidae

(Zetterstedt, 1838)

AB781654-BCB3-5CF1-B280-3BA2B2238F3B

####### Literature reference.

• spring of Bijela rijeka, Plitvice Lakes NP (1) • spring of Crna rijeka, Plitvice Lakes NP (4) (Ivković et al. 2015).

###### Paracladius conversus

Taxon classificationAnimaliaDipteraChironomidae

(Walker, 1856)

BA2439DB-2038-542C-862A-72E68140865C

####### Literature reference.

• Lake Prošće, Plitvice Lakes NP (7) • Lake Kozjak, Plitvice Lakes NP (17) (Kostić-Brnek and Brnek-Kostić 1971).

####### Remark.

Mentioned as *Paratrichocladius inserpens* Pankratova, 1970 in Kostić-Brnek and Brnek-Kostić (1971), a synonym of *P. conversus* in Moller Pillot (2013).

###### Parametriocnemus stylatus

Taxon classificationAnimaliaDipteraChironomidae

(Spaerck, 1923)

32BC2406-F9FF-517E-8B21-B1BA81A0A177

####### Literature references.

• Plitvički Ljeskovac, Plitvice Lakes NP (3) (Matoničkin 1987) • tufa barrier Burget-Kozjak, Plitvice Lakes NP (15) (Matoničkin et al. 1971, Matoničkin 1987) • tufa barrier Gavanovac-Kaluđerovac, Plitvice Lakes NP (20) • Stream Plitvica, Plitvice Lakes NP (24) (Matoničkin 1987).

####### New records.

• 11 larvae; Lake Kozjak, Plitvice Lakes NP (17); 18 Jul. 2018 • 2♂; tufa barrier Kozjak-Milanovac, Plitvice Lakes NP (18); 30 Nov. 2009; M. Ivković leg.

####### Remark.

Mentioned as *Limnophyes transcaucasicus* Chernovskij, 1949 in Matoničkin et al. (1971) and Matoničkin (1987), a synonym of *P. stylatus* in Moller Pillot (2013).

###### Parametriocnemus cf. stylatus sensu

Taxon classificationAnimaliaDipteraChironomidae

Moller Pillot, 2013

9928C8EF-BBC3-5C8A-AE77-8BD046432E13

####### Literature reference.

• spring of Crna rijeka, Plitvice Lakes NP (4) (Ivković et al. 2015).

###### Paraphaenocladius cf. exagitans sensu

Taxon classificationAnimaliaDipteraChironomidae

Moller Pillot, 2013

091E1A71-3CC9-51CA-9AD3-A83D1B7BD4F0

####### Literature reference.

• spring of Crna rijeka, Plitvice Lakes NP (4) (Ivković et al. 2015).

###### Paraphaenocladius impensus

Taxon classificationAnimaliaDipteraChironomidae

(Walker, 1856)

72C9ADC2-CB0B-5E70-B6EE-1FE7D7F23182

####### Literature reference.

• spring of Bijela rijeka, Plitvice Lakes NP (1) (Ivković et al. 2015).

###### Paraphaenocladius cf. irritus sensu

Taxon classificationAnimaliaDipteraChironomidae

Moller Pillot, 2013

6374D05F-0974-5CC4-938A-D6AB02265B2F

####### Literature reference.

• spring of Crna rijeka, Plitvice Lakes NP (4) (Ivković et al. 2015).

###### Paratrichocladius skirwithensis

Taxon classificationAnimaliaDipteraChironomidae

(Edwards, 1929)

9A79A9FA-7F4D-53CA-93C9-D6FDEBEF99AD

####### Literature reference.

• spring of Bijela rijeka, Plitvice Lakes NP (1) • spring of Crna rijeka, Plitvice Lakes NP (4) (Ivković et al. 2015).

###### Psectrocladius (Psectrocladius) barbimanus

Taxon classificationAnimaliaDipteraChironomidae

(Edwards, 1929)

9842176A-60F7-5D5B-9317-3A0FEFE45634

####### Literature reference.

• tufa barrier Labudovac, Plitvice Lakes NP (8) (Matoničkin et al. 1971, Matoničkin 1987) • Crna Rijeka by the bridge, Plitvice Lakes NP (6) • Stream Plitvica, Plitvice Lakes NP (24) (Matoničkin 1987).

###### Psectrocladius (Psectrocladius) psilopterus

Taxon classificationAnimaliaDipteraChironomidae

(Kieffer, 1906)

49DFA0B8-E2BD-5EE4-9BBB-A42437B6D962

####### Literature reference.

• Crna Rijeka by the bridge, Plitvice Lakes NP (6) (Matoničkin 1987).

###### Rheocricotopus effusus

Taxon classificationAnimaliaDipteraChironomidae

(Walker, 1856)

24F0760D-B489-5606-A809-502908DAD481

####### Literature reference.

• spring of Bijela rijeka, Plitvice Lakes NP (1) • spring of Crna rijeka, Plitvice Lakes NP (4) (Ivković et al. 2015).

###### Synorthocladius semivirens

Taxon classificationAnimaliaDipteraChironomidae

(Kieffer, 1909)

1E53DD43-8034-5819-930E-8400326B7F89

####### Literature reference.

• spring of Bijela rijeka, Plitvice Lakes NP (1) (Ivković et al. 2015).

####### New record.

• 1 larva; Lake Prošće, Plitvice Lakes NP (7); 26 Jul. 2019.

###### Thienemannia gracilis

Taxon classificationAnimaliaDipteraChironomidae

Kieffer, 1909

3579455D-D460-59F9-9A9D-3377DA6702D6

####### Literature reference.

• spring of Crna rijeka, Plitvice Lakes NP (4) (Ivković et al. 2015).

###### Tvetenia veralli

Taxon classificationAnimaliaDipteraChironomidae

(Edwards, 1929)

9016265A-9D78-5066-9E97-B1DB5FA5B52A

####### Literature reference.

• spring of Bijela rijeka, Plitvice Lakes NP (1) (Ivković et al. 2015).

##### Subfamily Tanypodinae

###### Ablabesmyia (Ablabesmyia) monilis

Taxon classificationAnimaliaDipteraChironomidae

(Linnaeus, 1758)

5883403F-3BD9-5FE5-B982-FCD9965DB13C

####### Literature reference.

• Lake Prošće, Plitvice Lakes NP (7) • Lake Kozjak, Plitvice Lakes NP (17) (Kostić-Brnek and Brnek-Kostić 1971).

####### Remark.

Mentioned as *Pentaneura monilis* Linnaeus, 1758 in Kostić-Brnek and Brnek-Kostić (1971).

###### Apsectrotanypus trifascipennis

Taxon classificationAnimaliaDipteraChironomidae

(Zetterstedt, 1838)

6CC14F8D-C278-5EE0-838B-1EF2EB4669AD

####### Literature reference.

• Lake Prošće, Plitvice Lakes NP (7) • Lake Kozjak, Plitvice Lakes NP (17) (Kostić-Brnek and Brnek-Kostić 1971).

####### Remark.

Mentioned as *Psectrotanypus trifascipennis* Zetterstedt, 1838 in Kostić-Brnek and Brnek-Kostić (1971) which is probably a misspelling.

###### * Labrundinia longipalpis

Taxon classificationAnimaliaDipteraChironomidae

(Goetghebuer, 1921)

E5F4D89D-AE54-52BA-967B-661A9CAF0C9E

####### New record.

• 9 larvae; Lake Prošće, Plitvice Lakes NP (7); 26 Jul. 2019.

###### Krenopelopia binotata

Taxon classificationAnimaliaDipteraChironomidae

(Wiedemann, 1817)

DFB8F68B-C564-5263-907F-E141C141F9F5

####### Literature reference.

• spring of Bijela rijeka, Plitvice Lakes NP (1) (Ivković et al. 2015).

###### Macropelopia cf. fehlmanni sensu

Taxon classificationAnimaliaDipteraChironomidae

Kieffer, 1912

46E9C450-05D4-5D61-9FB8-6157C4A8CDD1

####### Literature reference.

• Lake Prošće, Plitvice Lakes NP (7) • Lake Kozjak, Plitvice Lakes NP (17) (Kostić-Brnek and Brnek-Kostić 1971).

###### Procladius (Holotanypus) choreus

Taxon classificationAnimaliaDipteraChironomidae

(Meigen, 1804)

F46AF496-485B-52A3-B9A4-1C86B14A1011

####### Literature reference.

• Lake Prošće, Plitvice Lakes NP (7) • Lake Kozjak, Plitvice Lakes NP (17) (Kostić-Brnek and Brnek-Kostić 1971).

###### Thienemannimyia carnea

Taxon classificationAnimaliaDipteraChironomidae

(Fabricius, 1805)

68168E54-6236-5717-9AEF-996799F24A41

####### Literature reference.

• Lake Prošće, Plitvice Lakes NP (7) • Lake Kozjak, Plitvice Lakes NP (17) (Kostić-Brnek and Brnek-Kostić 1971).

####### Remark.

Mentioned as *Pentaneura carnea* Fabricius, 1805 in Kostić-Brnek and Brnek-Kostić (1971).

#### Family Dixidae

##### Dixa dilatata

Taxon classificationAnimaliaDipteraDixidae

Strobl, 1900

E6819CE9-0152-5751-825F-EBD9956A5035

###### Literature reference.

• Stream Sartuk, Plitvice Lakes NP (23) (Ivković and Ivanković 2019).

##### Dixa maculata

Taxon classificationAnimaliaDipteraDixidae

Meigen, 1818

D070D6BD-29DF-5E45-A450-F6100507ED68

###### Literature reference.

• upper reach of Bijela rijeka, Plitvice Lakes NP (2) • upper reach of Crna rijeka, Plitvice Lakes NP (5) • tufa barrier Labudovac, Plitvice Lakes NP (8) • tufa barrier Kozjak-Milanovac, Plitvice Lakes NP (18) • tufa barrier Novakovića Brod, Plitvice Lakes NP (22) (Ivanković et al. 2019).

##### Dixa nebulosa

Taxon classificationAnimaliaDipteraDixidae

Meigen, 1830

C3CB7468-FD6C-5F7C-8580-DF112382086B

###### Literature reference.

• upper reach of Bijela rijeka, Plitvice Lakes NP (2) • tufa barrier Labudovac, Plitvice Lakes NP (8) (Ivanković et al. 2019) • Lake Kozjak, Plitvice Lakes NP (17) (Ivković and Ivanković 2019).• tufa barrier Kozjak- Milanovac, Plitvice Lakes NP (18) • tufa barrier Novakovića Brod, Plitvice Lakes NP (22) • Stream Plitvica, Plitvice Lakes NP (24) • Korana Village, Plitvice Lakes NP (25) (Ivanković et al. 2019).

##### Dixa nubilipennis

Taxon classificationAnimaliaDipteraDixidae

Curtis, 1832

E1E3AAD8-B79F-5E2B-A46C-59046BF2EE81

###### Literature reference.

• Korana Village, Plitvice Lakes NP (Ivanković et al. 2019) (25).

##### Dixa puberula

Taxon classificationAnimaliaDipteraDixidae

Loew, 1849

58060D95-036B-5BDF-9608-4B47A3700125

###### Literature reference.

• spring of Bijela rijeka stream, Plitvice Lakes NP (1) • upper reach of Bijela rijeka, Plitvice Lakes NP (2) (Ivanković et al. 2019) • spring of Crna rijeka, Plitvice Lakes NP (4) (Ivković et al. 2015) • upper reach of Crna rijeka, Plitvice Lakes NP (5) • Crna rijeka by the bridge, Plitvice Lakes NP (6) (Ivanković et al. 2019) • tufa barrier Labudovac, Plitvice Lakes NP (8) • tufa barrier Kozjak- Milanovac, Plitvice Lakes NP (18) • tufa barrier Novakovića Brod, Plitvice Lakes NP (22) • Stream Plitvica, Plitvice Lakes NP (24) (Ivanković et al. 2019, Ivković and Ivanković 2019) • Korana Village, Plitvice Lakes NP (25) (Ivanković et al. 2019).

##### Dixa submaculata

Taxon classificationAnimaliaDipteraDixidae

Edwards, 1920

2058B08C-EE33-5E01-833E-FF4EBC3E5778

###### Literature reference.

• spring of Bijela rijeka, Plitvice Lakes NP (1) • upper reach of Bijela rijeka, Plitvice Lakes NP (2) • upper reach of Crna rijeka, Plitvice Lakes NP (5) • Crna rijeka by the bridge, Plitvice Lakes NP (6) • tufa barrier Kozjak-Milanovac, Plitvice Lakes NP (18) • tufa barrier Novakovića Brod, Plitvice Lakes NP (22) (Ivanković et al. 2019).

##### Dixella aestivalis

Taxon classificationAnimaliaDipteraDixidae

(Meigen, 1818)

254947C7-C4FB-5D79-B0CF-E00758EB3A75

###### Literature reference.

• Lake Okrugljak, Plitvice Lakes NP (9) • Riječica, Plitvice Lakes NP (16) (Matoničkin 1987).

##### Dixella amphibia

Taxon classificationAnimaliaDipteraDixidae

(De Geer, 1776)

C0F61D81-3852-58D5-8434-79BA8D82B3A5

###### Literature reference.

• tufa barrier Labudovac, Plitvice Lakes NP (8) (Matoničkin 1987).

###### New record.

• 1 larva; lake Kozjak, Plitvice Lakes NP (17); 11 Jul. 2019.

##### * Dixella autumnalis

Taxon classificationAnimaliaDipteraDixidae

(Meigen, 1838)

7E69F4E3-3E1C-5F81-9F57-004234263752

###### New record.

• 1 larva; lake Kozjak, Plitvice Lakes NP (17); 18 Jul. 2018.

#### Family Empididae

##### Subfamily Clinocerinae

###### Clinocera stagnalis

Taxon classificationAnimaliaDipteraEmpididae

(Haliday, 1833)

21DB4238-A63C-5BAD-B628-93A3F97DBBED

####### Literature reference.

• spring of Bijela rijeka, Plitvice Lakes NP (1) • spring of Crna rijeka, Plitvice Lakes NP (4) • Stream Plitvica, Plitvice Lakes NP (24) (Ivković et al. 2010, 2012a).

####### New record.

• 1♂; tufa barrier Labudovac, Plitvice Lakes NP (8); 26 Jul. 2013; M. Ivković leg.

###### Clinocera wesmaeli

Taxon classificationAnimaliaDipteraEmpididae

(Macquart, 1835)

C6650BC1-F024-5414-8721-0028034C2DBE

####### Literature reference.

• spring of Bijela rijeka, Plitvice Lakes NP (1) (Ivković et al. 2010, 2012a).

###### Dolichocephala guttata

Taxon classificationAnimaliaDipteraEmpididae

(Haliday, 1833)

CDB3172F-F8B7-5637-A7B9-58E90EADF88E

####### Literature reference.

• spring of Bijela rijeka, Plitvice Lakes NP (1) • upper reach of Bijela rijeka, Plitvice Lakes NP (2) • spring of Crna rijeka, Plitvice Lakes NP (4) • upper reach of Crna rijeka, Plitvice Lakes NP (5) • Korana Village, Plitvice Lakes NP (25) (Ivković et al. 2010, 2012a).

###### Dolichocephala ocellata

Taxon classificationAnimaliaDipteraEmpididae

(Costa, 1854)

B1B8821F-8B5C-55EA-AA43-B339060DA2D6

####### Literature reference.

• spring of Bijela rijeka, Plitvice Lakes NP (1) • upper reach of Bijela rijeka, Plitvice Lakes NP (2) (Ivković et al. 2010, 2012a).

###### Kowarzia barbatula

Taxon classificationAnimaliaDipteraEmpididae

(Mik, 1880)

D5C8CE79-EB70-5EB0-B39E-B2BC70E3EF00

####### Literature reference.

• spring of Bijela rijeka, Plitvice Lakes NP (1) • spring of Crna rijeka, Plitvice Lakes NP (4) • upper reach of Crna rijeka, Plitvice Lakes NP (5) • Crna rijeka by the bridge, Plitvice Lakes NP (6) • tufa barrier Labudovac, Plitvice Lakes NP (8) • tufa barrier Kozjak-Milanovac, Plitvice Lakes NP (18) • Stream Plitvica, Plitvice Lakes NP (24) (Ivković et al. 2010, 2012a).

###### Kowarzia bipunctata

Taxon classificationAnimaliaDipteraEmpididae

(Haliday, 1833)

BF9404AB-46F4-5C04-AAEF-DC5CC38F2A68

####### Literature reference.

• upper reach of Bijela rijeka, Plitvice Lakes NP (2) (Ivković et al. 2010).

###### Wiedemannia aquilex

Taxon classificationAnimaliaDipteraEmpididae

(Loew, 1869)

49227207-B89D-5149-9112-FCD1307DA120

####### Literature references.

• spring of Bijela rijeka, Plitvice Lakes NP (1) • upper reach of Bijela rijeka, Plitvice Lakes NP (2) • spring of Crna rijeka, Plitvice Lakes NP (4) • upper reach of Crna rijeka, Plitvice Lakes NP (5) • Crna rijeka by the bridge, Plitvice Lakes NP (6) (Ivković et al. 2010, 2012a).

###### Wiedemannia lamellata

Taxon classificationAnimaliaDipteraEmpididae

(Loew, 1869)

2E9B3F9D-718F-5C0E-9E94-64BB877D2F40

####### Literature references.

• spring of Bijela rijeka, Plitvice Lakes NP (1) • tufa barrier Novakovića Brod, Plitvice Lakes NP (22) • Stream Plitvica, Plitvice Lakes NP (24) • Korana Village, Plitvice Lakes NP (25) (Ivković et al. 2010, 2012a).

###### Wiedemannia zetterstedti

Taxon classificationAnimaliaDipteraEmpididae

(Fallén, 1826)

DD0CA69A-6960-50A1-B38E-796B144939CF

####### Literature reference.

• spring of Bijela rijeka, Plitvice Lakes NP (1) (Ivković et al. 2010).

##### Subfamily Hemerodromiinae

###### Chelifera concinnicauda

Taxon classificationAnimaliaDipteraEmpididae

Collin, 1927

9C1F6129-2A5D-5E88-B042-95456EEC3180

####### Literature references.

• Crna rijeka by the bridge, Plitvice Lakes NP (6) • tufa barrier Labudovac, Plitvice Lakes NP (8) • tufa barrier Kozjak-Milanovac, Plitvice Lakes NP (18) • tufa barrier Novakovića Brod, Plitvice Lakes NP (22) • Stream Plitvica, Plitvice Lakes NP (24) (Ivković et al. 2010, 2012a) • Korana Village, Plitvice Lakes NP (25) (Horvat 1990, Ivković et al. 2010, 2012a).

###### Chelifera flavella

Taxon classificationAnimaliaDipteraEmpididae

(Zetterstedt, 1838)

6C8DAD32-B79A-56D1-801F-11050BBAE9C4

####### Literature references.

• spring of Bijela rijeka, Plitvice Lakes NP (1) • upper reach of Bijela rijeka, Plitvice Lakes NP (2) • spring of Crna rijeka, Plitvice Lakes NP (4) • upper reach of Crna rijeka, Plitvice Lakes NP (5) (Ivković et al. 2010, 2012a).

###### Chelifera precabunda

Taxon classificationAnimaliaDipteraEmpididae

Collin, 1961

A988FC28-CA38-519F-AFC7-050248103433

####### Literature references.

• spring of Bijela rijeka, Plitvice Lakes NP (1) • upper reach of Bijela rijeka, Plitvice Lakes NP (2) • spring of Crna rijeka, Plitvice Lakes NP (4) • upper reach of Crna rijeka, Plitvice Lakes NP (5) • Crna rijeka by the bridge, Plitvice Lakes NP (6) (Ivković et al. 2010, 2012a).

###### Chelifera precatoria

Taxon classificationAnimaliaDipteraEmpididae

(Fallén, 1816)

771B2DE7-44E2-5DED-8564-D239B67CE25E

####### Literature references.

• spring of Bijela rijeka, Plitvice Lakes NP (1) • upper reach of Bijela rijeka, Plitvice Lakes NP (2) • spring of Crna rijeka, Plitvice Lakes NP (4) (Ivković et al. 2010, 2012a).

###### Chelifera pyrenaica

Taxon classificationAnimaliaDipteraEmpididae

Vaillant, 1981

9C98ABD7-0199-5A0D-9F8D-7F93370A13B8

####### Literature references.

• Crna rijeka by the bridge, Plitvice Lakes NP (6) • tufa barrier Kozjak-Milanovac, Plitvice Lakes NP (18) • tufa barrier Novakovića Brod, Plitvice Lakes NP (22) • Stream Plitvica, Plitvice Lakes NP (24) • Korana Village, Plitvice Lakes NP (25) (Ivković et al. 2010, 2012a).

###### Chelifera siveci

Taxon classificationAnimaliaDipteraEmpididae

Wagner, 1984

67055991-0C4B-50A5-911D-25F87D4927F8

####### Literature references.

• spring of Bijela Rijeka, Plitvice Lakes NP (1) • upper reach of Bijela rijeka, Plitvice Lakes NP (2) • spring of Crna Rijeka, Plitvice Lakes NP (4) • upper reach of Crna rijeka, Plitvice Lakes NP (5) • Crna rijeka by the bridge, Plitvice Lakes NP (6) • Stream Plitvica, Plitvice Lakes NP (24) (Ivković et al. 2010, 2012a).

###### Chelifera stigmatica

Taxon classificationAnimaliaDipteraEmpididae

(Schiner, 1862)

5215CF54-6634-5300-88D7-711C6F7A9CCC

####### Literature references.

• tufa barrier Labudovac, Plitvice Lakes NP (8) • tufa barrier Kozjak-Milanovac, Plitvice Lakes NP (18) • tufa barrier Novakovića Brod, Plitvice Lakes NP (22) • Stream Plitvica, Plitvice Lakes NP (24) (Ivković et al. 2010, 2012a).

###### Chelifera trapezina

Taxon classificationAnimaliaDipteraEmpididae

(Zetterstedt, 1838)

1BF23556-4AE3-5239-A8AB-43EE829B622C

####### Literature references.

• spring of Bijela rijeka, Plitvice Lakes NP (1) • upper reach of Bijela rijeka, Plitvice Lakes NP (2) • spring of Crna rijeka, Plitvice Lakes (4) • upper reach of Crna rijeka, Plitvice Lakes NP (5) • Crna rijeka by the bridge, Plitvice Lakes NP (6) • Stream Plitvica, Plitvice Lakes NP (24) • Korana Village, Plitvice Lakes NP (25) (Ivković et al. 2010, 2012a).

###### Hemerodromia laudatoria

Taxon classificationAnimaliaDipteraEmpididae

Collin, 1927

957BA7DF-D47A-5AB2-A9D1-D2F7D187F3A0

####### Literature references.

• Crna rijeka by the bridge, Plitvice Lakes NP (6) • Lake Prošće, Plitvice Lakes NP (7) (Ivković et al. 2010, 2012a).

###### Hemerodromia melangyna

Taxon classificationAnimaliaDipteraEmpididae

Collin, 1927

0E5ABF0D-1270-5146-B80C-B885F3140CD1

####### Literature references.

• tufa barrier Labudovac, Plitvice Lakes NP (8) • tufa barrier Novakovića Brod, Plitvice Lakes NP (22) • Stream Plitvica, Plitvice Lakes NP (24) (Ivković et al. 2010, 2012a) • Korana Village, Plitvice Lakes NP (25) (Horvat 1990, Ivković et al. 2010, 2012a).

####### New record.

• 1♂, 43♀; tufa barrier Kozjak-Milanovac, Plitvice Lakes NP (18); 29 Jun. 2015.

###### Hemerodromia oratoria

Taxon classificationAnimaliaDipteraEmpididae

(Fallén, 1816)

247F6D38-78B9-579C-988B-3855D978568F

####### Literature references.

• tufa barrier Novakovića Brod, Plitvice Lakes NP (22) • Stream Plitvica, Plitvice Lakes NP (24) (Ivković et al. 2010, 2012a).

###### Hemerodromia raptoria

Taxon classificationAnimaliaDipteraEmpididae

Meigen, 1830

BE53DCC6-4210-5602-96DC-1A6F4C96EB77

####### Literature references.

• Lake Kozjak, Plitvice Lakes NP (17) (Ivković et al. 2010, 2012a) • tufa barrier Kozjak-Milanovac, Plitvice Lakes NP (18) (Ivković et al. 2013b).

###### Hemerodromia unilineata

Taxon classificationAnimaliaDipteraEmpididae

Zetterstedt, 1842

E3B59BCB-3964-55EE-9FA5-EA8BFD83C517

####### Literature references.

• tufa barrier Labudovac, Plitvice Lakes NP (8) • tufa barrier Kozjak-Milanovac, Plitvice Lakes NP (18) • tufa barrier Novakovića Brod, Plitvice Lakes NP (22) • Stream Plitvica, Plitvice Lakes NP (24) • Korana Village, Plitvice Lakes NP (25) (Ivković et al. 2010, 2012a).

#### Family Limoniidae

##### Subfamily Chioneinae

###### Ellipteroides (Ellipteroides) lateralis

Taxon classificationAnimaliaDipteraLimoniidae

(Macquart, 1835)

5E93522D-9D29-52D1-8776-C1236BEC22E6

####### Literature reference.

• tufa barrier Kozjak-Milanovac, Plitvice Lakes NP (18) (Kolcsár et al. 2015).

###### Gonomyia (Gonomyia) tenella

Taxon classificationAnimaliaDipteraLimoniidae

(Meigen, 1818)

887DCDAA-C9AF-518E-8A23-CD2110C9039B

####### Literature reference.

• spring of Bijela rijeka, Plitvice Lakes NP (1) • Korana Village, Plitvice Lakes NP (25) (Kolcsár et al. 2015).

###### Molophilus (Molophilus) bifidus

Taxon classificationAnimaliaDipteraLimoniidae

Goetghebuer, 1920

87F2B8EA-62E5-579E-A732-F737B2C7FBDE

####### Literature reference.

• spring of Bijela rijeka, Plitvice Lakes NP (1) (Kolcsár et al. 2015).

###### Molophilus (Molophilus) repentinus

Taxon classificationAnimaliaDipteraLimoniidae

Starý, 1971

150515A9-75EA-5EB7-A0B8-86923EDFC614

####### Literature reference.

• Korana Village, Plitvice Lakes NP (25) (Kolcsár et al. 2015).

###### Ormosia (Oreophila) bergrothi

Taxon classificationAnimaliaDipteraLimoniidae

(Strobl, 1895)

4531046C-0271-5D3A-A5C8-ECD5F3245CD2

####### Literature reference.

• spring of Crna rijeka, Plitvice Lakes NP (4) (Kolcsár et al. 2015).

###### Rhabdomastix (Rhabdomastix) edwardsi

Taxon classificationAnimaliaDipteraLimoniidae

Tjeder, 1967

7021B698-19F4-5F54-8591-7DD134FF4C80

####### Literature reference.

• Korana Village, Plitvice Lakes NP (25) (Kolcsár et al. 2015).

###### Rhypholophus phryganopterus

Taxon classificationAnimaliaDipteraLimoniidae

Kolenati, 1860

B2CB51D1-19A0-5065-BF8A-A0FD38185E5E

####### Literature reference.

• spring of Crna rijeka, Plitvice Lakes NP (4) (Kolcsár et al. 2015).

##### Subfamily Limnophilinae

###### Eloeophila apicata

Taxon classificationAnimaliaDipteraLimoniidae

(Loew, 1871)

A1D46072-7702-57D4-9C82-986C272023E3

####### Literature reference.

• upper reach of Bijela rijeka, Plitvice Lakes NP (2) (Kolcsár et al. 2015).

###### Eloeophila maculata

Taxon classificationAnimaliaDipteraLimoniidae

(Meigen, 1804)

7D481999-D07F-569B-B913-D391D18CF335

####### Literature reference.

• upper reach of Bijela rijeka, Plitvice Lakes NP (2) • Crna rijeka by the bridge, Plitvice Lakes NP (6) (Kolcsár et al. 2015).

###### Epiphragma (Epiphragma) ocellare

Taxon classificationAnimaliaDipteraLimoniidae

(Linnaeus, 1760)

1A6F3674-0968-53B8-9C62-313E0C3EEDA3

####### Literature reference.

• spring of Crna rijeka, Plitvice Lakes NP (4) (Kolcsár et al. 2015).

####### Remark.

This species is not aquatic. Larvae develop in forests, woodlands, larvae associated with woody debris.

###### Hexatoma (Eriocera) chirothecata

Taxon classificationAnimaliaDipteraLimoniidae

(Scopoli, 1763)

62721BAB-0315-56D1-BF2D-CCBC173F9A85

####### Literature reference.

• tufa barrier Novakovića Brod, Plitvice Lakes NP (22) • Korana Village, Plitvice Lakes NP (25) (Kolcsár et al. 2015).

###### Paradelphomyia senilis

Taxon classificationAnimaliaDipteraLimoniidae

(Haliday, 1833)

2CECCDE0-FBE2-5580-8875-16B73C905EFB

####### Literature reference.

• upper reach of Bijela rijeka, Plitvice Lakes NP (2) • spring of Crna rijeka, Plitvice Lakes NP (4) (Kolcsár et al. 2015).

##### Subfamily Limoniinae

###### Antocha (Antocha) vitripennis

Taxon classificationAnimaliaDipteraLimoniidae

(Meigen, 1830)

16F577E3-7A4C-5B42-92F7-0BE01A7918C5

####### Literature reference.

• tufa barrier Kozjak-Milanovac, Plitvice Lakes NP (18) • tufa barrier Novakovića Brod, Plitvice Lakes NP (22) • Korana Village, Plitvice Lakes NP (25) (Kolcsár et al. 2015).

###### Dicranomyia (Dicranomyia) chorea

Taxon classificationAnimaliaDipteraLimoniidae

(Meigen, 1818)

080E5B59-2834-5EC3-98C1-2F45E1ED8B92

####### Literature reference.

• spring of Bijela rijeka, Plitvice Lakes NP (1) • upper reach of Bijela rijeka, Plitvice Lakes NP (2) • tufa barrier Kozjak-Milanovac, Plitvice Lakes NP (18) • Stream Plitvica, Plitvice Lakes NP (24) • Korana Village, Plitvice Lakes NP (25) (Kolcsár et al. 2015).

####### Remark.

Larvae associated with rotting woody debris, but sometimes also reared from semiaquatic habitats; larvae possibly feeding in partially submerged wood.

###### Dicranomyia (Dicranomyia) didyma

Taxon classificationAnimaliaDipteraLimoniidae

(Meigen, 1804)

98DF9E35-55D4-5034-AC9D-2180C97B2797

####### Literature reference.

• spring of Bijela rijeka, Plitvice Lakes NP (1) • upper reach of Bijela rijeka, Plitvice Lakes NP (2) • tufa barrier Kozjak-Milanovac, Plitvice Lakes NP (18) • Stream Plitvica, Plitvice Lakes NP (24) (Kolcsár et al. 2015).

###### Dicranomyia (Dicranomyia) imbecilla

Taxon classificationAnimaliaDipteraLimoniidae

Lackschewitz, 1941

3015A999-BC76-5888-BCB7-972E15EC89FD

####### Literature reference.

• spring of Bijela rijeka, Plitvice Lakes NP (1) • tufa barrier Kozjak-Milanovac, Plitvice Lakes NP (18) (Kolcsár et al. 2015).

###### Dicranomyia (Dicranomyia) mitis

Taxon classificationAnimaliaDipteraLimoniidae

(Meigen, 1830)

EAAE2F6C-EF9E-5372-B4C1-8B0CB21F711B

####### Literature reference.

• upper reach of Bijela rijeka, Plitvice Lakes NP (2) (Kolcsár et al. 2015).

####### Remarks.

Mentioned as Dicranomyia (Dicranomyia) mitis (Meigen, 1830) complex by Kolcsár et al. (2015). After re-identification of specimens by Kolcsár L.-P., using the identification key published by Starý and Stubbs (2015), it was confirmed that the specimens belong to Dicranomyia (Dicranomyia) mitis (Meigen, 1830).

###### Limonia hercegovinae

Taxon classificationAnimaliaDipteraLimoniidae

(Strobl, 1898)

2DC905E5-2FFF-507F-AE0C-43F57D066A3F

####### Literature reference.

• upper reach of Bijela rijeka, Plitvice Lakes NP (2) • spring of Crna rijeka, Plitvice Lakes NP (4) (Kolcsár et al. 2015).

####### Remark.

Larvae unknown, but perhaps not associated with aquatic habitats as other *Limonia* species have terrestrial larvae.

###### Lipsothrix nobilis

Taxon classificationAnimaliaDipteraLimoniidae

Loew, 1873

D68D57B9-F6DA-5889-9E15-AA3AF8E2F610

####### Literature reference.

• Crna rijeka by the bridge, Plitvice Lakes NP (6) • tufa barrier Labudovac, Plitvice Lakes NP (8) (Kolcsár et al. 2015).

#### Family Muscidae

##### Subfamily Coenosiinae

###### Limnophora croatica

Taxon classificationAnimaliaDipteraMuscidae

Pont & Ivković, 2013

C16C77CB-440E-5ED3-95B7-BB203DB80BF5

####### Literature references.

• spring of Bijela rijeka, Plitvice Lakes NP (1) (Pont and Ivković 2013, Ivković and Pont 2016) • spring of Crna Rijeka, Plitvice Lakes NP (4) (Pont and Ivković 2013) • tufa barrier Labudovac, Plitvice Lakes NP (8) (Pont and Ivković 2013, Ivković and Pont 2016) • Stream Plitvica, Plitvice Lakes NP (24) • Korana Village, Plitvice Lakes NP (25) (Pont and Ivković 2013).

###### Limnophora olympiae

Taxon classificationAnimaliaDipteraMuscidae

Lyneborg, 1965

5698E78C-09C8-5204-A8D3-19830CB4869D

####### Literature reference.

• spring of Bijela rijeka, Plitvice Lakes NP (1) • tufa barrier Kozjak-Milanovac, Plitvice Lakes NP (18) (Pont and Ivković 2013, Ivković and Pont 2016).

###### Limnophora pulchriceps

Taxon classificationAnimaliaDipteraMuscidae

(Loew, 1860)

3B6C0FE3-FC5E-5A0C-861D-213DA82D50DA

####### Literature references.

• tufa barrier Labudovac, Plitvice Lakes NP (8) • tufa barrier Kozjak-Milanovac, Plitvice Lakes NP (18) (Pont and Ivković 2013, Ivković and Pont 2016) • tufa barrier Novakovića Brod, Plitvice Lakes NP (22) • Stream Plitvica, Plitvice Lakes NP (24) (Pont and Ivković 2013).

###### Limnophora riparia

Taxon classificationAnimaliaDipteraMuscidae

(Fallén, 1824)

2144160F-1ED9-5AE2-9BD3-271EBFD88CB3

####### Literature references.

• spring of Bijela rijeka, Plitvice Lakes NP (1) • upper reach of Bijela rijeka, Plitvice Lakes NP (2) (Pont and Ivković 2013) • tufa barrier Labudovac, Plitvice Lakes NP (8) (Matoničkin et al. 1971, Pont and Ivković 2013, Ivković and Pont 2016) • tufa barrier Batinovac-Galovac, Plitvice Lakes NP (12) • tufa barrier Galovac-Milino, Plitvice Lakes NP (13) • tufa barrier Burget-Kozjak, Plitvice Lakes NP (15) (Matoničkin et al. 1971) • tufa barrier Kozjak-Milanovac, Plitvice Lakes NP (18) (Pont and Ivković 2013, Ivković and Pont 2016) • tufa barrier Milke Trnine, Plitvice Lakes NP (19) (Matoničkin et al. 1971) • tufa barrier Novakovića Brod, Plitvice Lakes NP (22) • Stream Plitvica, Plitvice Lakes NP (24) • Korana Village, Plitvice Lakes NP (25) (Pont and Ivković 2013).

###### Limnophora setinerva

Taxon classificationAnimaliaDipteraMuscidae

Schnabl, 1911

81CB66C4-227E-5C59-8C53-B2AA7B5BFE75

####### Literature references.

• spring of Bijela rijeka, Plitvice Lakes NP (1) • upper reach of Bijela rijeka, Plitvice Lakes NP (2) (Pont and Ivković 2013) • tufa barrier Labudovac, Plitvice Lakes NP (8) • tufa barrier Kozjak-Milanovac, Plitvice Lakes NP (18) (Pont and Ivković 2013, Ivković and Pont 2016) • Stream Plitvica, Plitvice Lakes NP (24) (Pont and Ivković 2013).

###### Limnophora tigrina

Taxon classificationAnimaliaDipteraMuscidae

(Am Stein, 1860)

688016FE-70D9-55A1-BB56-FBD59CDE9776

####### Literature reference.

• Korana Village, Plitvice Lakes NP (25) (Pont and Ivković 2013).

###### Limnophora triangula

Taxon classificationAnimaliaDipteraMuscidae

(Fallén, 1825)

3280BFB0-F31D-583F-ABCC-7B48C316D39E

####### Literature reference.

• tufa barrier Kozjak-Milanovac, Plitvice Lakes NP (18) (Ivković and Pont 2016) • Korana Village, Plitvice Lakes NP (25) (Pont and Ivković 2013).

###### Lispe tentaculata

Taxon classificationAnimaliaDipteraMuscidae

(De Geer, 1776)

CB0C4188-8037-5C3B-9506-C5C56B04FE43

####### Literature reference.

• Korana Village, Plitvice Lakes NP (25) (Ivković and Pont 2015).

###### Lispocephala brachialis

Taxon classificationAnimaliaDipteraMuscidae

(Rondani, 1877)

BF74A91A-26AB-57EC-A2F0-54E6BB89579F

####### Literature reference.

• tufa barrier Kozjak-Milanovac, Plitvice Lakes NP (18) (Ivković and Pont 2015).

###### Lispocephala spuria

Taxon classificationAnimaliaDipteraMuscidae

(Zetterstedt, 1838)

614C2883-E2E2-5327-B05B-C284C8C46E31

####### Literature reference.

• spring of Bijela rijeka, Plitvice Lakes NP (1) (Ivković and Pont 2015).

#### Family Pediciidae

##### Subfamily Pediciinae

###### Dicranota (Dicranota) bimaculata

Taxon classificationAnimaliaDipteraPediciidae

(Schummel, 1829)

BC0C4A13-7547-5BEF-89BD-AC4BA5A5749F

####### Literature reference.

• spring of Bijela rijeka, Plitvice Lakes NP (1) (Kolcsár et al. 2015).

###### Dicranota (Paradicranota) pavida

Taxon classificationAnimaliaDipteraPediciidae

(Haliday, 1833)

F006DE3F-BB08-506E-B6DF-2B2E341ADE88

####### Literature reference.

• upper reach of Bijela rijeka, Plitvice Lakes NP (2) (Kolcsár et al. 2015).

###### Pedicia (Amalopis) occulta

Taxon classificationAnimaliaDipteraPediciidae

(Meigen, 1830)

4DE18219-F6C5-5599-80F1-4EF60128F8D2

####### Literature reference.

• spring of Bijela rijeka, Plitvice Lakes NP (1) • upper reach of Bijela rijeka, Plitvice Lakes NP (2) • spring of Crna rijeka, Plitvice Lakes NP (4) • Crna rijeka by the bridge, Plitvice Lakes NP (6) (Kolcsár et al. 2015).

###### Tricyphona (Tricyphona) immaculata

Taxon classificationAnimaliaDipteraPediciidae

(Meigen, 1804)

083D0FD8-39AB-5AB9-9F8B-98D3B1417D43

####### Literature reference.

• spring of Bijela rijeka, Plitvice Lakes NP (1) • upper reach of Bijela rijeka, Plitvice Lakes NP (2) • spring of Crna rijeka, Plitvice Lakes NP (4) • tufa barrier Labudovac, Plitvice Lakes NP (8) (Kolcsár et al. 2015).

#### Family Psychodidae

##### Subfamily Sycoracinae

###### Sycorax feuerborni

Taxon classificationAnimaliaDipteraPsychodidae

Jung, 1954

E9E6EA3C-168C-5DD1-9AE2-42998A488E5F

####### Literature reference.

• spring of Crna Rijeka, Plitvice Lakes NP (4) (Ivković et al. 2015).

###### Sycorax tonnoiri

Taxon classificationAnimaliaDipteraPsychodidae

Jung, 1953

C60EDF74-8AD7-5CBA-BCDD-09B64BA4E855

####### Literature reference.

• spring of Crna Rijeka, Plitvice Lakes NP (4) (Kvifte et al. 2013, Ivković et al. 2015).

##### Subfamily Psychodinae

###### Berdeniella keroveci

Taxon classificationAnimaliaDipteraPsychodidae

Kvifte, Ivković & Klarić, 2013

F454712F-963C-5931-A9AF-FF7D276290B4

####### Literature reference.

• spring of Bijela rijeka, Plitvice Lakes NP (1) • spring of Crna Rijeka, Plitvice Lakes NP (4) (Kvifte et al. 2013, Ivković et al. 2015).

###### Pericoma blandula

Taxon classificationAnimaliaDipteraPsychodidae

Eaton, 1893

031B6CE1-D5B4-5FCC-8255-1538ECFF3FFE

####### Literature reference.

• tufa barrier Labudovac, Plitvice Lakes NP (8) (Kvifte et al. 2013).

###### Pericoma miljenkoi

Taxon classificationAnimaliaDipteraPsychodidae

Kvifte & Ivković, 2018

CB2F82EF-A073-5933-92F2-17CC3CEA6BA7

####### Literature reference.

• tufa barrier Labudovac, Plitvice Lakes NP (8) • tufa barrier Kozjak-Milanovac, Plitvice Lakes NP (18) (Kvifte and Ivković 2018).

###### Pericoma pseudocalcilega

Taxon classificationAnimaliaDipteraPsychodidae

Krek, 1972

81B26EC8-4B7F-5A69-8BB7-544D2C166639

####### Literature reference.

• tufa barrier Labudovac, Plitvice Lakes NP (8) (Kvifte et al. 2013).

###### Psychoda (Logima) albipennis

Taxon classificationAnimaliaDipteraPsychodidae

Zetterstedt, 1850 complex

55CA66F4-222D-56E6-90F6-F261C591AC79

####### Literature reference.

• spring of Bijela rijeka, Plitvice Lakes NP (1) (Kvifte et al. 2013).

###### Psychoda (Psychodocha) gemina

Taxon classificationAnimaliaDipteraPsychodidae

(Eaton, 1904)

88DEC171-BB90-5BF8-A63B-672FD70565ED

####### Literature reference.

• spring of Crna Rijeka, Plitvice Lakes NP (4) (Kvifte et al. 2013, Ivković et al. 2015).

###### Jungiella valachia

Taxon classificationAnimaliaDipteraPsychodidae

(Vaillant, 1963)

375A427F-4BAC-533D-8D1E-03018274B079

####### Literature reference.

• spring of Bijela rijeka, Plitvice Lakes NP (1) (Kvifte et al. 2013, Ivković et al. 2015).

#### Family Scathophagidae

##### Subfamily Scathophaginae

###### * Acanthocnema latipennis

Taxon classificationAnimaliaDipteraPsychodidae

Becker, 1894

A2F327BF-7094-5F8D-8BEC-9031612EFB78

####### New records.

• 4♂; spring of Bijela rijeka, Plitvice Lakes NP (1); 26 Jul. 2016; M. Ivković leg. • 2♂; tufa barrier Kozjak-Milanovac, Plitvice Lakes NP (18); 29 Apr. 2015; M. Ivković leg.

#### Family Simuliidae

##### Subfamily Simuliinae

###### Prosimulium tomosvaryi

Taxon classificationAnimaliaDipteraPsychodidae

(Enderlein, 1921)

9B97DF10-CCE3-52F6-8190-29C4934968D1

####### Literature references.

• Stream Plitvica, Plitvice Lakes NP (24) • Korana Village, Plitvice Lakes NP (25) (Ivković et al. 2012b, 2014).

###### Simulium (Eusimulium) angustipes

Taxon classificationAnimaliaDipteraPsychodidae

Edwards, 1915

589F1997-3327-5C89-B866-BDEFF9C00769

####### Literature reference.

• tufa barrier Labudovac, Plitvice Lakes NP (8) • tufa barrier Kozjak-Milanovac, Plitvice Lakes NP (18) • tufa barrier Novakovića Brod, Plitvice Lakes NP (22) • Korana Village, Plitvice Lakes NP (25) (Ivković et al. 2012b, 2014).

###### Simulium (Eusimulium) rubzovianum

Taxon classificationAnimaliaDipteraPsychodidae

(Sherban, 1961)

EA0C9106-5195-5530-9B09-32EC39645B0C

####### Literature reference.

• tufa barrier Labudovac, Plitvice Lakes NP (8) • tufa barrier Kozjak-Milanovac, Plitvice Lakes NP (18) • tufa barrier Novakovića Brod, Plitvice Lakes NP (22) • Stream Plitvica, Plitvice Lakes NP (24) (Ivković et al. 2016).

###### Simulium (Nevermannia) angustitarse

Taxon classificationAnimaliaDipteraPsychodidae

(Lundström, 1911)

361456AD-F826-5F02-89D3-D4A9000AD5D3

####### Literature reference.

• upper reach of Bijela rijeka, Plitvice Lakes NP (2) (Ivković et al. 2016).

###### Simulium (Nevermannia) costatum

Taxon classificationAnimaliaDipteraPsychodidae

Friederichs, 1920

4748DB72-2F0E-5ECF-8291-8932485995B2

####### Literature references.

• spring of Bijela Rijeka, Plitvice Lakes NP (1) • upper reach of Bijela rijeka, Plitvice Lakes NP (2) • upper reach of Crna rijeka, Plitvice Lakes NP (5) • Crna rijeka by the bridge, Plitvice Lakes NP (6) • tufa barrier Labudovac, Plitvice Lakes NP (8) • tufa barrier Kozjak-Milanovac, Plitvice Lakes NP (18) • tufa barrier Novakovića Brod, Plitvice Lakes NP (22) • Stream Plitvica, Plitvice Lakes NP (24) • Korana Village, Plitvice Lakes NP (25) (Ivković et al. 2012b, 2014).

###### Simulium (Simulium) monticola

Taxon classificationAnimaliaDipteraPsychodidae

Friederichs, 1920

3BC596D9-DA46-58F0-9680-74917B42FE92

####### Literature references.

• upper reach of Bijela rijeka, Plitvice Lakes NP (2) • Crna rijeka by the bridge, Plitvice Lakes NP (6) • tufa barrier Labudovac, Plitvice Lakes NP (8) (Ivković et al. 2012b, 2014).

####### New record.

• 7♂; tufa barrier Kozjak-Milanovac, Plitvice Lakes NP (18); 28 Jun. 2007; M. Ivković leg.

###### Simulium (Simulium) ornatum

Taxon classificationAnimaliaDipteraPsychodidae

Meigen, 1818 (complex)

52B1B217-4143-5EA3-BB99-DA68E583FA8D

####### Literature references.

• tufa barrier Kozjak-Milanovac, Plitvice Lakes NP (18) • Stream Plitvica, Plitvice Lakes NP (24) • Korana Village, Plitvice Lakes NP (25) (Ivković et al. 2012b, 2014).

####### New records.

• 4♂; tufa barrier Labudovac, Plitvice Lakes NP (8); 29 May 2009; M. Ivković leg. • 3♂; same site; 30 Jun. 2009; M. Ivković leg.

###### Simulium (Simulium) trifasciatum

Taxon classificationAnimaliaDipteraPsychodidae

Curtis, 1839

44D611C1-3239-5A57-9025-1FEC7BC9BB1F

####### Literature references.

• tufa barrier Labudovac, Plitvice Lakes NP (8) • tufa barrier Kozjak-Milanovac, Plitvice Lakes NP (18) (Ivković et al. 2012b, 2014).

###### Simulium (Simulium) tuberosum

Taxon classificationAnimaliaDipteraPsychodidae

(Lundström, 1911)

11EA5F96-54B1-556F-ADB6-B4C3D8587AC9

####### Literature references.

• tufa barrier Labudovac, Plitvice Lakes NP (8) • tufa barrier Novakovića Brod, Plitvice Lakes NP (22) • Korana Village, Plitvice Lakes NP (25) (Ivković et al. 2012b, 2014).

###### Simulium (Simulium) variegatum

Taxon classificationAnimaliaDipteraPsychodidae

Meigen, 1818

322E55B6-6684-5874-B665-EDE294B27AFC

####### Literature references.

• tufa barrier Labudovac, Plitvice Lakes NP (8) • tufa barrier Novakovića Brod, Plitvice Lakes NP (22) • Stream Plitvica, Plitvice Lakes NP (24) • Korana Village, Plitvice Lakes NP (25) (Ivković et al. 2012b, 2014).

###### Simulium (Trichodagmia) auricoma

Taxon classificationAnimaliaDipteraPsychodidae

Meigen, 1818

9DE2D9AD-F50C-5643-952D-2CA1DF8FBEF7

####### Literature references.

• tufa barrier Kozjak-Milanovac, Plitvice Lakes NP (18) • tufa barrier Novakovića Brod, Plitvice Lakes NP (22) • Korana Village, Plitvice Lakes NP (25) (Ivković et al. 2012b, 2014).

###### Simulium (Wilhelmia) pseudequinum

Taxon classificationAnimaliaDipteraPsychodidae

Séguy, 1921

AE4EDA02-B721-59B7-AA54-2E9A88CAB941

####### Literature references.

• tufa barrier Kozjak-Milanovac, Plitvice Lakes NP (18) • tufa barrier Novakovića Brod, Plitvice Lakes NP (22) (Ivković et al. 2012b, 2014).

####### Remark.

Formerly this was misidentified as Simulium (Wilhelmia) equinum (Linnaeus, 1758) in Ivković et al. (2012, 2014, 2016).

#### Family Stratiomyidae

##### * Oxycera pardalina

Taxon classificationAnimaliaDipteraPsychodidae

Meigen, 1822

DAB1EB86-FBE7-5EAB-A0E8-6DA175BA23C1

###### New records.

• 1 larva; spring of Bijela rijeka, Plitvice Lakes NP (1); 30 May 2008; M. Ivković leg. • 6 larvae; upper reach of Bijela rijeka, Plitvice Lakes NP (2); 29 May 2007; M. Ivković leg. • 1♀; same site; 26 Jul. 2010; M. Ivković leg. • 1 larva; upper reach of Crna rijeka, Plitvice Lakes NP (5); 30 Apr. 2007; M. Ivković leg. • 4 larvae; same site; 29 May 2007; M. Ivković leg. • 1 larva, 1♀; same site; 30 Jun. 2007; M. Ivković leg. • 2 larvae; same site; 30 Apr. 2008; M. Ivković leg. • 1 larva; same site; 30 May 2008; M. Ivković leg. • 1♀; tufa barrier Labudovac, Plitvice Lakes NP (8); 30 Jun. 2008; M. Ivković leg. • 1♂; same site; 30 Jun. 2011; M. Ivković leg. • 1♀; same site; 28 Jun. 2012; M. Ivković leg.

##### * Oxycera limbata

Taxon classificationAnimaliaDipteraPsychodidae

Loew, 1862

ECB27779-8C33-54D0-B430-F4A76F0A8CD9

###### New records.

• 1♀; tufa barrier Labudovac, Plitvice Lakes NP (8); 25 Jul. 2011; M. Ivković leg. • 3♂; Korana Village, Plitvice Lakes NP (25); 29 Jun. 2007; M. Ivković leg.

##### * Oxycera turcica

Taxon classificationAnimaliaDipteraPsychodidae

Ustuner & Hasbenli, 2004

F2F11AE7-AA40-5D75-98BD-7FABF7E28E02

###### New records.

• 3♂, 2♀; Korana Village, Plitvice Lakes NP (25); 29 Jun. 2007; M. Ivković leg. • 1♂, 2♀; same site; 26 Jul. 2007; M. Ivković leg.

##### * Nemotelus pantherinus

Taxon classificationAnimaliaDipteraPsychodidae

(Linnaeus, 1758)

8BAC8E33-144E-56B2-89DE-16451CD18E16

###### New record.

• 1♀; upper reach of Bijela rijeka, Plitvice Lakes NP (1); 24 Jul. 2009; M. Ivković leg.

##### * Oplodontha viridula

Taxon classificationAnimaliaDipteraPsychodidae

(Fabricius, 1775)

E0F1C82F-BE1E-5499-966C-0E1E87F342CD

###### New record.

• 1♀; tufa barrier Labudovac, Plitvice Lakes NP (8); 26 Jul. 2010; M. Ivković leg.

#### Family Tabanidae

##### Subfamily Chrysopsinae

###### Chrysops caecutiens

Taxon classificationAnimaliaDipteraTabanidae

(Linnaeus, 1758)

B25E33ED-860D-5785-986B-09C5FDBA21BF

####### Literature reference.

• Plitvički Ljeskovac, Plitvice Lakes NP (3) (Krčmar et al. 2008).

###### Chrysops viduatus

Taxon classificationAnimaliaDipteraTabanidae

(Fabricius, 1794)

F67AF0F4-2578-582A-8CA2-D2D55ADD6BF4

####### Literature reference.

• Plitvički Ljeskovac, Plitvice Lakes NP (3) (Krčmar et al. 2008).

#### Family Tipulidae

##### Subfamily Tipulinae

###### Tipula (Savtshenkia) rufinarufina

Taxon classificationAnimaliaDipteraTipulidae

Meigen, 1818

39D18816-4A7F-5D2B-85F6-C525A5B541D0

####### Literature reference.

• spring of Bijela rijeka, Plitvice Lakes NP (1) (Ivković et al. 2015).

####### New records.

• 2♂; spring of Bijela rijeka, Plitvice Lakes NP (1); 1 Oct. 2009; M. Ivković leg. • 1♂; spring of Bijela rijeka, Plitvice Lakes NP (1); 2 Nov. 2011; M. Ivković leg. • 1♀; spring of Bijela rijeka, Plitvice Lakes NP (1); 27 Jun. 2013; M. Ivković leg.

### Species richness and assemblage composition

In total, 157 species and 7 taxa of aquatic Diptera (Table 2) belonging to 13 families, collected from 25 sites (Table 1, Figure 1) are recorded in the Plitvice Lakes NP, with twelve species new for the dipteran fauna of the National Park. New species belonging to the family Chironomidae are *Labrundinia longipalpis* (Goetghebuer, 1921), *Nilothauma brayi* (Goetghebuer, 1921), *Potthastia longimanus* Kieffer, 1922, Polypedilum (Polypedilum) nubeculosum (Meigen, 1804) and *Tanytarsus brundini* Lindeberg, 1963; to the family Dixidae is *Dixella autumnalis* (Meigen, 1838), and Scathophagidae*Acanthocnema latipennis* Becker, 1894. New species found in the Plitvice Lakes NP belonging to Stratiomyidae family are *Oxycera pardalina* Meigen, 1822, *Oxycera limbata* Loew, 1862, *Oxycera turcica* Ustuner & Hasbenli, 2004, *Nemotelus pantherinus* (Linnaeus, 1758), and *Oplodontha viridula* (Fabricius, 1775).

**Table 2.** Aquatic Diptera at different types of karstic habitats in National Park Plitvice Lakes.

Species/Habitat type	Spring	Stream	Tufa barrier	Lake
** Athericidae **				
*Ibisia marginata* (Fabricius, 1781)		•	•	
** Chironomidae **				
Ablabesmyia (Ablabesmyia) monilis (Linnaeus, 1758)				•
*Acricotopus lucens* (Zetterstedt, 1850)				•
*Apsectrotanypus trifascipennis* (Zetterstedt, 1838)				•
*Brillia bifida* (Kieffer, 1909)	•			
*Brillia longifurca* Kieffer, 1921				•
*Chaetocladius dentiforceps* (Edwards, 1929)	•			
*Chaetocladius melaleucus* (Meigen, 1818)	•			
*Corynoneura lobata* Edwards, 1924	•			
Cricotopus (Cricotopus) bicinctus (Meigen, 1818)				•
Cricotopus (Cricotopus) fuscus (Kieffer, 1909)		•		
Cryptochironomus (Cryptochironomus) albofasciatus (Staeger, 1839)				•
Diamesa (Diamesa) thomasi Serra-Tosio, 1970	•			
Diamesa (Diamesa) tonsa (Haliday in Walker, 1856)	•			
*Dicrotendipes nervosus* (Staeger, 1839)				•
*Einfeldia dissidens* (Walker, 1856)				•
Endochironomus cf. dispar sensu Moller Pillot, 2009				•
*Epoicocladius ephemerae* (Kieffer, 1924)	•			
*Eukiefferiella devonica* (Edwards, 1929)	•			
*Eukiefferiella ilkleyensis* (Edwards, 1929)	•			
*Eukiefferiella minor* (Edwards, 1929)	•			
*Eukiefferiella gracei* (Edwards, 1929)	•			
*Harinischia fuscimanus* Kieffer, 1921				•
*Heterotrissocladius marcidus* (Walker, 1856)				•
*Krenopelopia binotata* (Wiedemann, 1817)	•			
*Labrundinia longipalpis* (Goetghebuer, 1921)				•
Limnophyes cf. minimus sensu Langton & Pinder, 2007	•			
*Limnophyes gurgicola* (Edwards, 1929)	•			
Macropelopia cf. fehlmanni sensu Kieffer, 1912				•
Metriocnemus cf. albolineatus sensu Langton & Pinder, 2007	•			
*Metriocnemus eurynothus* (Holmgren, 1883)	•			
*Metriocnemus intergerivus* Sæther, 1995	•			
*Micropsectra notescens* (Walker, 1856)	•			
*Micropsectra uva* Giłka, Zakrzewska, Baranov & Dominiak, 2013	•			
*Microtendipes pedellus* (De Geer, 1776)				•
*Microtendipes tarsalis* (Walker, 1856)				•
*Monodiamesa bathyphila* (Kieffer, 1918)				•
*Nilothauma brayi* (Goetghebuer, 1921)				•
Orthocladius (Mesorthocladius) frigidus (Zetterstedt 1838)	•			
*Paracladius conversus* (Walker, 1856)				•
*Paracladopelma camptolabis* (Kieffer, 1913)				•
Parametriocnemus cf. stylatus sensu Moller Pillot, 2013	•			
*Parametriocnemus stylatus* (Spaerck, 1923)		•	•	
Paraphaenocladius cf. exagitans sensu Moller Pillot, 2013	•			
*Paraphaenocladius impensus* (Walker, 1856)	•			
Paraphaenocladius cf. irritus sensu Moller Pillot, 2013	•			
*Paratanytarsus lauterborni* (Kieffer, 1909)				•
*Paratendipes albimanus* (Meigen, 1818)				•
*Paratrichocladius skirwithensis* (Edwards, 1929)	•			
*Phaenopsectra flavipes* (Meigen 1818)	•			•
Polypedilum (Pentapedilum) exsectum (Kieffer, 1916)				•
Polypedilum (Polypedilum) nubeculosum (Meigen, 1804)				•
Polypedilum (Tripodura) scalaenum (Schrank, 1803)				•
*Potthastia longimanus* Kieffer, 1922				•
Procladius (Holotanypus) choreus (Meigen, 1804)				•
*Prodiamesa olivacea* (Meigen, 1818)	•		•	•
Psectrocladius (Psectrocladius) barbimanus (Edwards, 1929)		•	•	
Psectrocladius (Psectrocladius) psilopterus (Kieffer, 1906)		•		
*Rheocricotopus effusus* (Walker, 1856)	•			
*Rheotanytarsus nigricauda* Fittkau, 1960	•			
*Rheotanytarsus pentapoda* (Kieffer, 1909)			•	
*Stempellina bausei* (Kieffer, 1911)		•	•	•
*Synorthocladius semivirens* (Kieffer, 1909)	•			
*Tanytarsus brundini* Lindeberg, 1963			•	
*Tanytarsus heusdensis* Goetghebuer, 1923				•
*Thienemannia gracilis* Kieffer, 1909	•			
*Thienemannimyia carnea* (Fabricius, 1805)				•
*Tvetenia veralli* (Edwards, 1929)	•			
*Zavrelia pentatoma* Kieffer & Bause, 1913				•
*Zavreliella marmorata* (van der Wulp, 1859)				•
** Dixidae **				
*Dixa dilatata* Strobl, 1900		•		
*Dixa maculata* Meigen, 1818		•	•	
*Dixa nebulosa* Meigen, 1830		•	•	•
*Dixa nubilipennis* Curtis, 1832		•		
*Dixa puberula* Loew, 1849	•	•	•	
*Dixa submaculata* Edwards, 1920	•	•	•	
*Dixella aestivalis* (Meigen, 1818)		•		•
*Dixella amphibia* (De Geer, 1776)			•	
*Dixella autumnalis* (Meigen, 1838)				•
** Empididae **				
*Chelifera concinnicauda* Collin, 1927		•	•	
*Chelifera flavella* (Zetterstedt, 1838)	•	•		
*Chelifera precabunda* Collin, 1961	•	•		
*Chelifera precatoria* (Fallén, 1816)	•	•		
*Chelifera pyrenaica* Vaillant, 1981		•	•	
*Chelifera siveci* Wagner, 1984	•	•		
*Chelifera stigmatica* (Schiner, 1862)		•	•	
*Chelifera trapezina* (Zetterstedt, 1838)	•	•		
*Clinocera stagnalis* (Haliday, 1833)	•	•		
*Clinocera wesmaeli* (Macquart, 1835)	•			
*Dolichocephala guttata* (Haliday, 1833)	•	•		
*Dolichocephala ocellata* (Costa, 1854)	•	•		
*Hemerodromia laudatoria* Collin, 1927		•		•
*Hemerodromia melangyna* Collin, 1927		•	•	
*Hemerodromia oratoria* (Fallén, 1816)		•	•	
*Hemerodromia raptoria* Meigen, 1830			•	•
*Hemerodromia unilineata* Zetterstedt, 1842		•	•	
*Kowarzia barbatula* (Mik, 1880)	•	•	•	
*Kowarzia bipunctata* (Haliday, 1833)		•		
*Wiedemannia aquilex* (Loew, 1869)	•	•		
*Wiedemannia lamellata* (Loew, 1869)	•	•	•	
*Wiedemannia zetterstedti* (Fallén, 1826)	•			
** Limoniidae **				
Antocha (Antocha) vitripennis (Meigen, 1830)		•	•	
Dicranomyia (Dicranomyia) chorea (Meigen, 1818)	•	•	•	
Dicranomyia (Dicranomyia) didyma (Meigen, 1804)	•	•	•	
Dicranomyia (Dicranomyia) imbecilla Lackschewitz, 1941	•		•	
Dicranomyia (Dicranomyia) mitis (Meigen, 1830) complex		•		
Ellipteroides (Ellipteroides) lateralis (Macquart, 1835)			•	
*Eloeophila apicata* (Loew, 1871)		•		
*Eloeophila maculata* (Meigen, 1804)		•		
Epiphragma (Epiphragma) ocellare (Linnaeus, 1760)	•			
Gonomyia (Gonomyia) tenella (Meigen, 1818)	•	•		
Hexatoma (Eriocera) chirothecata (Scopoli, 1763)		•	•	
*Limonia hercegovinae* (Strobl, 1898)	•	•		
*Lipsothrix nobilis* Loew, 1873		•	•	
Molophilus (Molophilus) bifidus Goetghebuer, 1920	•			
Molophilus (Molophilus) repentinus Starý, 1971		•		
Ormosia (Oreophila) bergrothi (Strobl, 1895)	•			
Paradelphomyia (Oxyrhiza) senilis (Haliday, 1833)	•	•		
Rhabdomastix (Rhabdomastix) edwardsi Tjeder, 1967		•		
*Rhypholophus phryganopterus* Kolenati, 1860	•			
** Muscidae **				
*Limnophora croatica* Pont & Ivković, 2013	•	•	•	
*Limnophora olympiae* Lyneborg, 1965	•		•	
*Limnophora pulchriceps* (Loew, 1860)		•	•	
*Limnophora riparia* (Fallén, 1824)	•	•	•	
*Limnophora setinerva* Schnabl, 1911	•	•	•	
*Limnophora tigrina* (Am Stein, 1860)		•		
*Limnophora triangula* (Fallén, 1825)		•	•	
*Lispe tentaculata* (De Geer, 1776)		•		
*Lispocephala brachialis* (Rondani, 1877)			•	
*Lispocephala spuria* (Zetterstedt, 1838)	•			
** Pediciidae **				
Dicranota (Dicranota) bimaculata (Schummel, 1829)	•			
Dicranota (Paradicranota) pavida (Haliday, 1833)		•		
Pedicia (Amalopis) occulta (Meigen, 1830)	•	•		
Tricyphona (Tricyphona) immaculata (Meigen, 1804)	•	•	•	
** Psychodidae **				
*Sycorax feuerborni* Jung, 1954	•			
*Sycorax tonnoiri* Jung, 1953	•			
*Berdeniella keroveci* Kvifte, Ivković & Klarić, 2013	•			
*Pericoma blandula* Eaton, 1893			•	
*Pericoma miljenkoi* Kvifte & Ivković, 2018			•	
*Pericoma pseudocalcilega* Krek, 1972			•	
Psychoda (Logima) albipennis Zetterstedt, 1850 complex	•			
Psychoda (Psychodocha) gemina (Eaton, 1904)	•			
*Jungiella valachia* (Vaillant, 1963)	•			
** Scathophagidae **				
*Acanthocnema latipennis* Becker, 1894	•		•	
** Simuliidae **				
*Prosimulium tomosvaryi* (Enderlein, 1921)		•		
Simulium (Eusimulium) angustipes Edwards, 1915		•	•	
Simulium (Eusimulium) rubzovianum (Sherban, 1961)		•	•	
Simulium (Nevermannia) angustitarse (Lundström, 1911)		•		
Simulium (Nevermannia) costatum Friederichs, 1920	•	•	•	
Simulium (Simulium) monticola Friederichs, 1920		•	•	
Simulium (Simulium) ornatum Meigen, 1818 complex		•	•	
Simulium (Simulium) trifasciatum Curtis, 1839			•	
Simulium (Simulium) tuberosum (Lundström, 1911)		•	•	
Simulium (Simulium) variegatum Meigen, 1818		•	•	
Simulium (Trichodagmia) auricoma Meigen, 1818		•	•	
Simulium (Wilhelmia) pseudequinum Séguy, 1921			•	
** Stratiomyidae **				
*Oxycera pardalina* Meigen, 1822	•	•	•	
*Oxycera limbata* Loew, 1862		•	•	
*Oxycera turcica* Ustuner & Hasbenli, 2004		•		
*Nemotelus pantherinus* (Linnaeus, 1758)		•		
*Oplodontha viridula* (Fabricius, 1775)			•	
** Tabanidae **				
*Chrysops caecutiens* (Linnaeus, 1758)		•		
*Chrysops viduatus* (Fabricius, 1794)		•		
** Tipulidae **				
Tipula (Savtshenkia) rufina rufina Meigen, 1818	•			
**Number of taxa**	**75**	**71**	**53**	**38**

**Figure 8.**
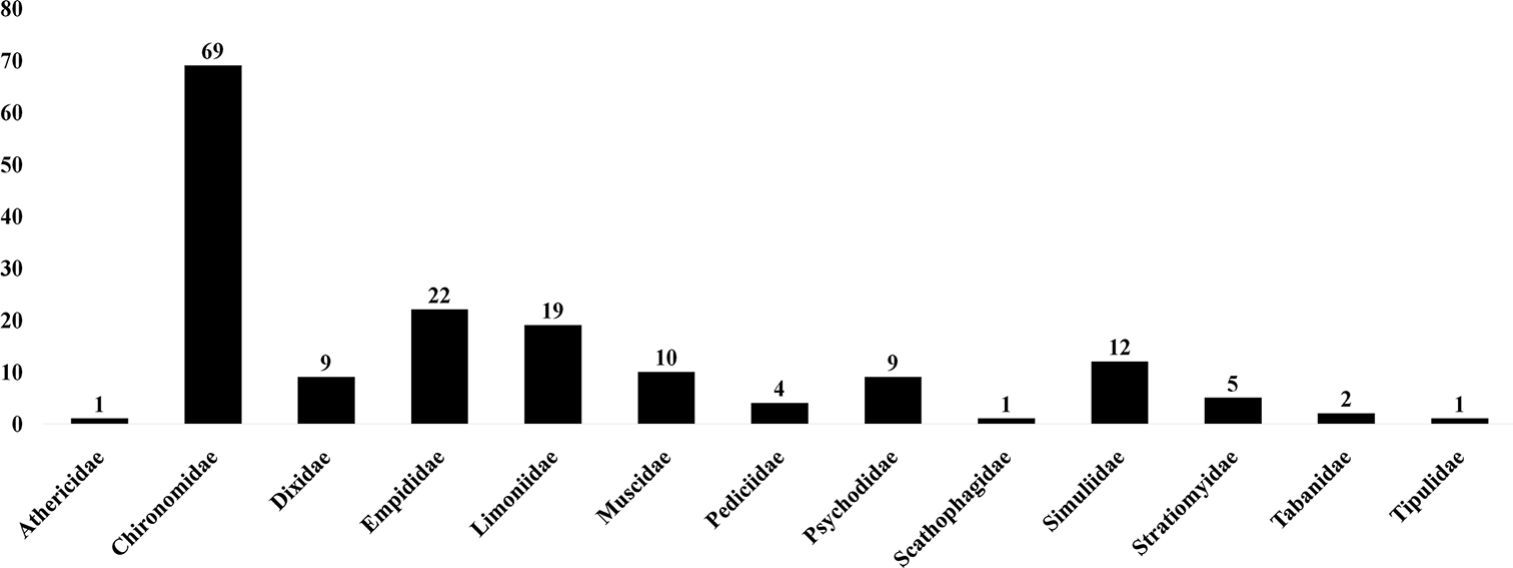
Species richness of Diptera families in Plitvice Lakes National Park.

Overall, the greatest species richness was recorded within the family Chironomidae, with 62 species (and additional seven taxa) recorded so far in Plitvice Lakes NP (Table 2, Figure 8). This was expected since the Chironomidae are an extremely diverse group with more than 8000 described species (Marshall 2012), and with many more undescribed or waiting to be discovered. Some chironomid species have been mentioned in the literature, but are not included in the formal list for various reasons that are considered here. The species *Micropsectra curvicornis* (Chernovskij, 1949) listed in Kostić-Brnek and Brnek-Kostić (1971) and *Thienemanniella flaviforceps* Kieffer, 1925, mentioned from Plitvice in Matoničkin (1987), are considered to be *nomina dubia* in Ashe and Cranston (1990), as well as in Ashe and O’Connor (2012). Ablabesmyia cf. tetrasticta could possibly be a misinterpretation of the name *Pelopia tetrasticta* Kieffer, 1916 and as such is considered a *nomen dubium* in the subfamily Tanypodinae (Ashe and O’Connor 2009). Furthermore, the species *Cricotopus latidentatus* (Chernovskij, 1949), published in Matoničkin (1987) and Matoničkin et al. (1971), is considered a questionable synonym within the genus *Cricotopus* according to the Ashe and O’Connor (2012). *Cricotopus filiformis* Edwards, mentioned in Matoničkin (1987), is considered an unidentifiable error since Edwards never described a chironomid species with “fili” in the name (Ashe and Cranston 1990). Chironomidae species that are part of the formal species list, but should be addressed with caution, are also considered. In Kostić-Brnek and Brnek-Kostić (1971), the authors did not clearly state whether their identifications of Endochironomus cf. dispar and Macropelopia cf. fehlmanni were based on adults or larvae. If the identifications were made based on adults, then these reports can be considered valid. On the other hand, if larvae were identified then these reports are doubtful records since these species cannot be identified based on larval morphology alone. The species listed in the formal species list as *Eukiefferiella ilkleyensis* (Edwards, 1929) is mentioned as *Plectrocladius eukiefferiella quadridentata* Chernovskij, 1949 in Matoničkin (1987). This could be a typing error since a genus *Plectrocladius* does not exist within the family Chironomidae. On the other hand, *Eukiefferiella quadridentata* Chernovskij, 1949 is a synonym pro parte of *E. ilkleyensis* (Moller Pillot 2013). If this is not a typing error, then this species name is an unidentifiable error.

Families following Chironomidae by number of species are Empididae, Limoniidae, Simuliidae and Muscidae (only aquatic species identified) with 22, 19, 12 and 10 species, respectively (Table 2, Figure 8). Dixidae and Psychodidae are both present with nine species, while Stratiomyidae, Pediciidae and Tabanidae are present with 5, 4 and 2 species, respectively (Table 2, Figure 8). Families with only one recorded species are Athericidae, Scathophagidae and Tipulidae (Table 2, Figure 8).

The families Dixidae, Empididae and Simuliidae have been dealt with in detail in previous publications (Ivković et al. 2010, 2012a, b, 2016; Ivanković et al. 2019; Ivković and Ivanković 2019) so the numbers presented here might be the final species numbers for Plitvice Lakes NP. For these particular families, only a few additional species might be recorded in the future studies. Other families of aquatic Diptera have been studied sporadically and, in many cases, only a few sites have been completely processed, such as Chironomidae in Kostić-Brnek and Brnek-Kostić (1971) and Ivković et al. (2015). Aquatic Diptera families that are present in the Plitvice Lakes NP but are still unidentified to the species level are Ceratopogonidae, Culicidae, Ephydridae, Sciomyzidae and Syrphidae.

Springs and streams have higher numbers of species recorded than tufa barriers and lakes (Table 2). One of the reasons for this is because the springs were studied in greater detail than other sites, especially when it comes to Chironomidae (Ivković et al. 2015). On the other hand, many aquatic Diptera families have in fact a higher diversity at spring habitats and the upper reaches of streams, such as Empididae, Psychodidae, Limoniidae, etc., because most environmental parameters at those sites remain constant (Ivković et al. 2010, 2012a, 2015; Kvifte et al. 2013; Kolcsár et al. 2015).

The species list of aquatic Diptera of Plitvice Lakes NP is still not complete since many families are either dealt with partially or not at all due to the lack of available experts. There is still a lot of work in front of us since we belive that about 250 species of aquatic Diptera can be expected in the unique karstic area of Plitvice Lakes NP.

## References

[B1] Adler PH Crosskey RW (2018) World Blackflies (Diptera: Simuliidae): a fully revised edition of the taxonomic and geographical inventory. http://entweb.clemson.edu/biomia/pdfs/blackflyinventory.pdf [Accessed 23 September 2019]

[B2] Adler PH Courtney GW (2019) Ecological and societal services of aquatic Diptera Insects 10, 70: 1–23. 10.3390/insects10030070 PMC6468872 30875770

[B3] Allegrucci G Carchini G Todisco V Convey P Sbordoni V (2006) A molecular phylogeny of Antarctic Chironomidae and its implications for biogeographical history. Polar Biology 29: 320–326. 10.1007/s00300-005-0056-7

[B4] Andersen T Cranston PS Epler JH (2013) The larvae of Chironomidae (Diptera) of the Holarctic Region – Keys and diagnoses. Insect Systematics and Evolution 66: 1–571.

[B5] Ashe P Cranston PS (1990) Chironomidae. In: Soós Á Papp L (Eds) Catalogue of Palaearctic Diptera.Elsevier Science Publishers, Amsterdam, The Netherlands, 113–355.

[B6] Ashe P O’Connor JP (2009) A World Catalogue of Chironomidae (Diptera). Part 1. Buchonomyiinae, Chilenomyiinae, Podonominae, Aphroteniinae, Tanypodinae, Usambaromyiinae, Diamesinae, Prodiamesinae and Telmatogetoninae. Irish Biogeographical Society and National Museum of Ireland, Dublin, 445 pp.

[B7] Ashe P O’Connor JP (2012) A World Catalogue of Chironomidae (Diptera). Part 2. Orthocladiinae. Irish Biogeographical Society and National Museum of Ireland, Dublin, 968 pp.

[B8] Baranov V Ivković M Willassen E (2013) First record of *Diamesa thomasi* Serra-Tosio, 1970, from Croatia. Chironomus Newsletter 26: 53–55. 10.5324/cjcr.v0i26.1623

[B9] Bitušík P (2000) Príručka na Určovanie Lariev Pakomárov (Diptera: Chironomidae) Slovenska. Čast’ I. Buchonomyinae, Diamesinae, Prodiamesinae a Orthocladiinae. Technická univerzita vo Zvolene, Zvolene, 133 pp.

[B10] Bitušík P Hamerlík L (2014) Príručka na Určovanie Lariev Pakomárov (Diptera: Chironomidae) Slovenska. Čast’ 2. Tanypodinae. Belianum. Vydavatel’stvo Univerzity Matej Bela v Banskej Bystrici, Banska Bystrica, 96 pp.

[B11] Collins LE Blackwell A (2000) The biology of *Toxorhynchites* mosquitoes and their potential as biocontrol agents. Biocontrol News and Information 21, 105N–116N.

[B12] Courtney GW Pape T Skevington JH Sinclair BJ (2017) Biodiversity of Diptera. In: Foottit RG Adler PH (Eds) Insect Biodiversity: Science and Society (Vol.I, 2^nd^ ed.). John Wiley and Sons, Chichester, 229–278. 10.1002/9781118945568.ch9

[B13] Disney RHL (1999) British Dixidae (Meniscus midges) and Thaumaleidae (Trickle midges): Keys with ecological notes. Freshwater Biological Association, Scientific Publication No. 56, Ambleside, 129 pp.

[B14] Engel EO (1938–1946) 28. Empididae. In: Linder E (Ed.) Die Fliegen der Palaearktischen Region, Bd 4(4). E. Schweizerbart’sche Verlagsbuchhandlung (Erwin Nägele), Stuttgart, 399 pp.

[B15] Ferrington LC Berg Jr. MB Coffman WP (2008) Chironomidae. In: Merritt RW Cummins KW Berg MB (Eds) An Introduction to the Aquatic Insects of North America (4th ed.). Kendall/Hunt Publishing Company, Dubuque, 847–989.

[B16] Giłka W Zakrzewska M Baranov VA Dominiak P (2013) Diagnostic clues for identification of selected species of the *Micropsectra atrofasciata* group, with description of *M. uva* sp. nov. from Croatia (Diptera: Chironomidae: Tanytarsini). Zootaxa 3702: 288–294. 10.11646/zootaxa.3702.3.6 26146725

[B17] Gorodkov KB (1988) Family Scatophagidae (Cordyluridae, Scatomyzidae, Scopeumatidae). In: Shtakel’berg AA Narchuk EP (Eds) Keys to the Insects of the European Part of the USSR in five Volumes, Flies, Fleas (2nd part).Nauka, Leningrad, 440–458.

[B18] Horvat B (1990) Aquatic dance flies of the subfamily Hemerodrominae (Diptera: Empididae) in Yugoslavia. Scopolia 20: 1–27.

[B19] Hölker F Vanni MJ Kuiper JJ Meile C Grossart H-P Stief P Adrian R Lorke A Dellwig O Brand A Hupfer M Mooij WM Nützmann G Lewandowski J (2015) Tube-dwelling invertebrates: tiny ecosystem engineers have large effects in lake ecosystems. Ecological Monographs 85: 333–351. 10.1890/14-1160.1

[B20] Ivanković L Ivković M Stanković I (2019) Perennial phenology patterns and ecological traits of Dixidae (Insecta, Diptera) in lotic habitats of a barrage lake system. Limnologica 76: 11–18. 10.1016/j.limno.2019.03.001

[B21] Ivković M Miliša M Mihaljević Z (2010) The aquatic dance flies fauna (Diptera, Empididae: Hemerodromiinae and Clinocerinae) of the Plitvice Lakes National Park. Natura Croatica 19: 133–139.

[B22] Ivković M Stanković Mičetić V Mihaljević Z (2012a) Emergence patterns and microhabitat preference of aquatic dance flies (Empididae; Clinocerinae and Hemerodromiinae) on a longitudinal gradient of barrage lake system. Limnologica 42: 43–49. 10.1016/j.limno.2011.07.003

[B23] Ivković M Kesić M Stloukalová V (2012b) Contribution to the knowledge of black fly (Diptera, Simuliidae) fauna at Plitvice lakes National Park. Natura Croatica 21: 263–268.

[B24] Ivković M Miliša M Previšić A Popijač A Mihaljević Z (2013a) Environmental factors and emergence patterns: case study of changes in hourly and daily emergence of aquatic insects at constant and variable water temperatures. International Review of Hydrobiology 98: 104–115. 10.1002/iroh.201301483

[B25] Ivković M Gračan R Horvat B (2013b) Croatian aquatic dance flies (Diptera: Empididae: Clinocerinae and Hemerodromiinae): species diversity, distribution and relationship to surrounding countries. Zootaxa 3686: 255–276. 10.11646/zootaxa.3686.2.7 26473217

[B26] Ivković M Kesić M Mihaljević Z Kudelá M (2014) Emergence patterns and ecological associations of hematophagous black flies along oligotrophic hydrosystem. Medical and Veterinary Entomology 28: 94–102. 10.1111/mve.12019 23834394

[B27] Ivković M Pont AC (2015) New records of Muscidae (Diptera) from Mediterranean countries. ZooKeys 496: 131–144. 10.3897/zookeys.496.9445 PMC4410160 25931958

[B28] Ivković M Miliša M Baranov V Mihaljević Z (2015) Environmental drivers of biotic traits and phenology patterns of Diptera assemblages in karst springs: The role of canopy uncovered. Limnologica 54: 44–57. 10.1016/j.limno.2015.09.001

[B29] Ivković M Kúdela M Kúdelová T (2016) Blackflies (Diptera: Simuliidae) in Croatia: species richness, distribution and relationship to surrounding countries. Zootaxa 4109: 16–30. 10.11646/zootaxa.4109.1.2 27394848

[B30] Ivković M Pont AC (2016) Long-time emergence patterns of *Limnophora* species (Diptera, Muscidae) in specific karst habitats: tufa barriers. Limnologica 61: 29–35. 10.1016/j.limno.2016.09.003

[B31] Ivković M Wahlberg E Previšić A (2019) Molecular phylogenetics and biogeography provide insights into the subgeneric classification of *Wiedemannia* Zetterstedt (Diptera: Empididae: Clinocerinae). Systematic Entomology 44: 559–570. 10.1111/syen.12340

[B32] Ivković M Ivanković L (2019) The genus *Dixa* (Diptera, Dixidae) in Croatian lotic habitats, with a checklist of species and relationships with the fauna of neighbouring countries. Zookeys 867: 45–54. 10.3897/zookeys.867.36613 31402838 PMC6684581

[B33] Krčmar S Jarić-Perkušić D Rajlić K (2008) Distribution of Tabanids from the genus *Chrysops* (Diptera: Tabanidae) in Croatia. Entomologica Croatica 12: 23–49.

[B34] Kolcsár L-P Ivković M Ternjej I (2015) New records of Limoniidae and Pediciidae (Diptera) from Croatia. ZooKeys 513: 23–37. 10.3897/zookeys.513.10066 PMC4524276 26257567

[B35] Kostić-Brnek LJ Brnek-Kostić A (1971) Nekoliko značajnijih vrsta u hironomidnoj fauni Plitvičkih jezera (Diptera, Chironomidae). Zbornik referata sa I simpozijuma biosistematičara Jugoslavije, 175–185.

[B36] Kvifte GM Ivković M Klarić A (2013) New records of moth flies (Diptera: Psychodidae) from Croatia, with the description of *Berdeniella keroveci* sp. nov. Zootaxa 3737: 57–67. 10.11646/zootaxa.3737.1.4 25112736

[B37] Kvifte GM Ivković M (2018) New species and records of the *Pericoma trifasciata* group from Croatia (Diptera: Psychodidae). Zootaxa 4486: 076–082. 10.11646/zootaxa.4486.1.5 30313767

[B38] Lackmann AR Butler MG (2018) Breaking the rule: Five larval instars in the podonomine midge *Trichotanypus alaskensis* Brundin from Barrow, Alaska. Journal of Limnology 77: 31–39. 10.4081/jlimnol.2018.1758

[B39] Langton PH Pinder LCV (2007a) Keys to the Adult Male Chironomidae of Britain and Ireland (Vol. 1). Freshwater biological association 64, 239 pp.

[B40] Langton PH Pinder LCV (2007b) Keys to the adult male Chironomidae of Britain and Ireland (Vol. 2). Freshwater biological association 64, 168 pp.

[B41] Larocque I Hall RI Grahn E (2001) Chironomids as indicators of climate change: A 100-lake training set from a subarctic region of northern Sweden (Lapland). Journal of Paleolimnology 26: 307–322. 10.1023/A:1017524101783

[B42] Marshall SA (2012) Flies. The Natural History and Diversity of Diptera. A Firefly Book, Richmond Hill, Ontario, 616 pp.

[B43] Matoničkin I (1987) Građa za limnofaunu krških voda tekućica Hrvatske, Plitvička jezera. Biosistematika 13: 25–35.

[B44] Matoničkin I Pavletić Z Tavčar V Krkač N (1971) Limnološka istraživanja reikotopa i fenomena protočne travertinizacije u Plitvičkim jezerima. The limnological investigations of reicotops and phenomenon of current travertinization in Plitvička jezera (Plitvice Lakes, Yugoslavia). Acta Biologica 7/1, Prirodoslovna istraživanja 40: 1–88.

[B45] Mihaljević Z Kerovec M Tavčar V Bukvić I (1998) Macroinvertebrate community on an artificial substrate in the Sava river: long-term changes in the community structure and water quality. Biologia 53: 611–620.

[B46] Moller Pillot HKM (2009) Chironomidae Larvae of the Netherlands and Adjacent Lowlands. Biology and Ecology of the Chironomini. KNNV Publishing, Zeist, The Netherlands, 270 pp.

[B47] Moller Pillot HKM (2013) Chironomidae Larvae of the Netherlands and Adjacent Lowlands. Biology and Ecology of aquatic Orthocladiinae. KNNV Publishing, Zeist, The Netherlands, 312 pp.

[B48] Oosterbroek P (2006) The European Families of the Diptera: Identification, diagnosis, biology. KNNV Publishing, Utrecht, The Netherlands, 205 pp.

[B49] Oosterbroek P (2019) Catalogue of the Craneflies of the World (Diptera, Tipuloidea: Pediciidae, Limoniidae, Cylindrotomidae, Tipulidae). http://ccw.naturalis.nl/index.php [accessed 31 October 2019]

[B50] Pape T (2009) Palaearctic Diptera – from Tundra to Desert. In: Pape T Bickel D Meier R (Eds) Diptera Diversity – Status, Challenges and Tools.Koninklijke Brill NV., 121–154. 10.1163/ej.9789004148970.I-459.27

[B51] Pape T Beuk P (2012) Fauna Europaea version 2017.06. https://www.faunaeur.org [accessed 20 September 2019]

[B52] Pont AC Ivković M (2013) The hunter-flies of Croatia (Diptera: Muscidae, genus *Limnophora* Robineau-Desvoidy). Journal of Natural History 47: 1069–1082. 10.1080/00222933.2012.750775

[B53] Reiss F Fittkau EF (1971) Taxonomie und Ökologie europäisch verbreiteter Tanytarsus-Arten (Chironomidae, Diptera). Archiv für Hydrobiologie 40 (1/2): 75–200.

[B54] Rozkošný R Kniepert F-W (2000) Insecta: Diptera: Stratomyidae, Tabanidae. Süβwasserfauna von Mitteleuropa 21/18, 19, Spectrum Akademischer Verlag GmbH, Heidelberg, 204 pp.

[B55] Sertić Perić M Dražina T Špoljar M Radanović I Primc B Habdija I (2014) Meiofauna constitute a considerable portion of invertebrate drift among moss-rich patches within a karst hydrosystem. Biologia 69: 363–380. 10.2478/s11756-013-0323-y

[B56] Spies M Saether OA (2013) Fauna Europaea: Chironomidae In: Pape T, Beuk P (2013) Fauna Europaea: Diptera Fauna Europaea version 2017.06. https://www.faunaeur.org [accessed 11 October 2019]

[B57] Srdoč D Horvatinčić N Obelić B Krajcar I Sliepčević A (1985) Procesi taloženja kalcita u krškim vodama s posebnim osvrtom na Plitvička jezera. Carsus Iugoslavie 11: 101–204.

[B58] Starý J (2019) Some notes on the genus *Paradelphomyia* Alexander (Diptera, Limoniidae). Dipterists Digest (2^nd^ series) 26: 57–60.

[B59] Starý J Stubbs AE (2015) Five species under Dicranomyia (Dicranomyia) mitis (Meigen, 1830) (Diptera, Limoniidae). Zootaxa 3964: 321–334. 10.11646/zootaxa.3964.3.2 26249442

[B60] Stilinović B Božičević S (1998) The Plitvice Lakes–a natural phenomenon in the middle of the Dinaric karst in Croatia. European Water Management 1: 15–24.

[B61] Šegota T Filipčić A (2003) Köppenova podjela klima i hrvatsko nazivlje. Geoadria 8: 17–23. 10.15291/geoadria.93

[B62] Thomas AGB (1997) DipteraRhagionidae and Athericidae, Snipe-flies. In: Nilsson A (Ed.) Aquatic Insects of North Europe (Vol.2). Apollo Books, Stenstrup, 311–320.

[B63] Vallenduuk HJ (2017) Chironomini larvae of western European lowlands (Diptera: Chironomidae). Keys with notes to the species. Lauterbornia 82: 1–216.

[B64] Walker IR (1987) Chironomidae (Diptera) in paleoecology. Quaternary Science Reviews 6: 29–40. 10.1016/0277-3791(87)90014-X

[B65] Werner D Pont AC (2003) Dipteran predators of simuliid blackflies: A worldwide review. Medical and Veterinary Entomology 17: 115–132. 10.1046/j.1365-2915.2003.00431.x 12823828

[B66] Wotton RS Malmqvist B Muotka T Larsson K (1998) Fecal pellets from a dense aggregation of suspension-feeders in a stream: An example of ecosystem engineering. Limnology and Oceanography 43: 719–725. 10.4319/lo.1998.43.4.0719

[B67] Yang D Zhang KY Yao G Zhang JH (2007) World catalog of Empididae (Insecta: Diptera). China Agricultural University Press, Beijing, 599 pp.

